# How Molecular Motors Are Arranged on a Cargo Is Important for Vesicular Transport

**DOI:** 10.1371/journal.pcbi.1002032

**Published:** 2011-05-05

**Authors:** Robert P. Erickson, Zhiyuan Jia, Steven P. Gross, Clare C. Yu

**Affiliations:** 1 Department of Physics and Astronomy, University of California, Irvine, Irvine, California, United States of America; 2 Department of Developmental and Cell Biology, University of California, Irvine, Irvine, California, United States of America; Technische Universität München, Germany

## Abstract

The spatial organization of the cell depends upon intracellular trafficking of cargos hauled along microtubules and actin filaments by the molecular motor proteins kinesin, dynein, and myosin. Although much is known about how single motors function, there is significant evidence that cargos *in vivo* are carried by multiple motors. While some aspects of multiple motor function have received attention, how the cargo itself —and motor organization on the cargo—affects transport has not been considered. To address this, we have developed a three-dimensional Monte Carlo simulation of motors transporting a spherical cargo, subject to thermal fluctuations that produce both rotational and translational diffusion. We found that these fluctuations could exert a load on the motor(s), significantly decreasing the mean travel distance and velocity of large cargos, especially at large viscosities. In addition, the presence of the cargo could dramatically help the motor to bind productively to the microtubule: the relatively slow translational and rotational diffusion of moderately sized cargos gave the motors ample opportunity to bind to a microtubule before the motor/cargo ensemble diffuses out of range of that microtubule. For rapidly diffusing cargos, the probability of their binding to a microtubule was high if there were nearby microtubules that they could easily reach by translational diffusion. Our simulations found that one reason why motors may be approximately 100 nm long is to improve their ‘on’ rates when attached to comparably sized cargos. Finally, our results suggested that to efficiently regulate the number of active motors, motors should be clustered together rather than spread randomly over the surface of the cargo. While our simulation uses the specific parameters for kinesin, these effects result from generic properties of the motors, cargos, and filaments, so they should apply to other motors as well.

## Author Summary

The spatial organization of living cells depends upon a transportation system consisting of molecular motor proteins that act like porters carrying cargos along filaments that are analogous to roads. The breakdown of this transportation system has been associated with neurodegenerative diseases such as Alzheimer's and Huntington's disease. In living cells, cargos are typically carried by multiple motors. While some aspects of multiple motor function have received attention, how the cargo itself affects transport has not been considered. To address this, we developed a three-dimensional computer simulation of motors transporting a spherical cargo subject to fluctuations produced when small molecules in the intracellular environment buffet the cargo. These fluctuations can cause the cargo to pull on the motors, slowing them down and making them detach from the filament (road). This effect increases as the cargo size and viscosity of the medium increase. We also found that the presence of the cargo helped the motors to bind to a filament before it drifted away. If other filaments were present, then the cargo could bind to one of them. Our results also indicated that it is better to group the motors on the cargo rather than spread them randomly over the surface.

## Introduction

Cells are highly organized, and much of this organization results from motors that move cargos along microtubules. The single-molecule properties of molecular motors are relatively well understood both experimentally and theoretically. With this as a starting point, we investigated how the presence of the cargo itself alters transport. Aside from exerting viscous drag, the cargo could in principle alter single-motor based transport both by changing the motors' diffusion and ability to contact the filament (a free motor diffuses very differently from a cargo-bound one), and also by exposing the motor to the random forces resulting from thermal fluctuations of the cargo which depend on the size of the cargo and the viscosity of the environment. Whether such effects are significant are investigated here.

Recent studies show that cargos *in vivo* are frequently moved by more than one microtubule-based motor [1], [2], [3], [4]. This raises the question of how multiple motors function together, the subject of recent theoretical and experimental work [1], [5], [6], [7]. *In vitro*, when more than one motor is actively hauling a cargo, the run length, i.e., the distance that the cargo travels along the microtubule before detaching, increases with the number of active motors. However, the presence of the cargo itself may be important when there are multiple motors. In addition to possibly changing the single-molecule's function, the cargo's size may alter the relationship between the total number of motors present and the number of motors actively engaged in transporting the cargo (assuming random motor organization on the cargo's surface). If motors are not randomly organized, details of this organization will also be important. How each of these factors contributes to overall transport is unknown.

To approach these problems requires a new theoretical framework: past studies simplified the problem using essentially one-dimensional models [5], [6], [8], [9] that had the motors attached to the cargo at a single point, with the cargo represented by a single point (though potentially experiencing viscous drag proportional to a specific diameter). Here we have developed a bone-fide three dimensional Monte Carlo simulation that allows us to directly investigate how the presence of the cargo itself affects single-motor driven transport and motor-microtubule attachment, as well as how the relationship between cargo size and the arrangement of motors on the cargo affects ultimate cargo motion, all within the context of a cargo experiencing random Brownian translational and rotational motion.

**Figure 1:**
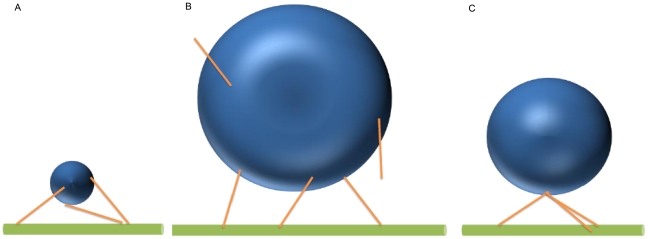
Kinesin motors (sticks) with a length of 110 nm are attached to cargos in various arrangements. (A) Motors on a cargo with diameter of 50 nm can easily reach the microtubule. (B) Motors on a cargo with a diameter of 500 nm have difficulty reaching the microtubule from most places on the cargo. (C) Motors attached to the cargo at the same point (South Pole) can easily reach the microtubule.

The attachment of motors to a cargo of finite size, rather than an idealized point mass, has a number of ramifications. First, the function of the motor(s) might be altered by the translational and rotational diffusion of the cargo; the larger the cargo, the more effect it has on the motors' diffusion, and thus, potentially, on the motors' ability to contact/interact with a microtubule. Second, when a motor is attached to both the microtubule and the cargo, it will feel instantaneous forces due to the cargo's thermal motion. These forces will depend on the cargo's size; and the random thermal ‘tugs’ from the cargo could slow the rate of travel of a motor and, in principle, induce the motor to detach from the filament. Third, there is a relationship between the cargo size, the total number of motors present, how they are arranged, and how many can be engaged. To illustrate this, imagine one cargo that is 50 nm in diameter, and another that is 500 nm in diameter. In the first case, even if the motors are randomly distributed on the cargo, because the length of an individual motor is more than 100 nm, all of those on the lower half of the cargo, and some on the upper half, will be able to reach a nearby microtubule (Figure 1A). In contrast for the 500 nm cargo, most motors will be unable to reach if they are randomly distributed on the cargo (Figure 1B). However, if all the motors were clumped at a single point, the size of the cargo essentially becomes irrelevant, because if one motor can reach, they all can (Figure 1C).

We thus set out to answer the following questions:

- How does a cargo affect the rate at which the motor(s) on the cargo bind to the microtubule?
  - For a single motor on a cargo, how does the presence of the cargo affect the motor's effective ‘on’ rate, i.e., the rate at which the motor binds to the microtubule?
  - How does the cargo's size and viscosity affect the probability that the cargo will bind to the microtubule before diffusing away?
  - Further, how does the length of the motor compared to the cargo size contribute to these properties?
  - What about the binding probability of a cargo with multiple motors?
  - How does the distance between microtubules affect the probability that a cargo with one or more motors will bind to a microtubule?
- Does the cargo's Brownian motion affect the motor's function as measured by its travel distance?
  - For a cargo with a single motor?
  - For a cargo with multiple motors?
- Number of engaged motors.
  - For randomly distributed motors, does doubling, say, the total number of motors on the cargo double the number of motors engaged in hauling the cargo along the microtubule?
  - What is the relationship between the cargo size, the viscosity, the number of motors present, the average number of motors actively engaged in transporting the cargo, and the cargos' mean travel distance?
- For motors “clustered” on the cargo, what is the relationship between cargo size, the viscosity, the size of the cluster, the number of motors in the cluster, and the mean travel distance?

We organized the presentation of our results according to these questions.

## Methods

### Monte Carlo simulations

To address these questions, we developed three-dimensional Monte Carlo simulations. Generally speaking, Monte Carlo is an approach to computer simulations in which an event A occurs with a certain probability P_A_ where 0≤P_A_≤1. In practice, during each time step, a random number *x* is generated with uniform probability between 0 and 1. If *x*≤P_A_, event *A* occurs; if *x*>P_A_, event *A* does not occur.

Our simulations were carried out as follows. We started with a three dimensional spherical cargo, subject to rotational and translational diffusion according to the equations presented below and in the Text S1. To this cargo, we attached kinesin motor(s) that are modeled as bungee cords, i.e., they behave as springs with a spring constant of 0.32 pN/nm [5], [10] when stretched beyond their relaxed length of 110 nm but produce no force when compressed. We started the simulation so that potentially one or more motors could bind to a cylindrical microtubule (25 nm diameter). The motors then moved the cargo along the microtubules, taking 8 nm steps. While technical details of the simulation are in the Text S1, the general idea is that at each time step *Δt*, we consider all motors present, calculate all forces acting upon them, and then ask what each of them does.

We start by describing how we simulate transport of a cargo with motors attached. Our basic algorithm is as follows. Consider one or more motors attached at random points to the cargo surface. The cargo is then suspended above the microtubule, with a well-defined separation distance between the bottom of the cargo and the top of the microtubule, and the motors are each given an opportunity to attach to the microtubule. If none do (either because none can reach, or because although they can reach, they stochastically are not able to attach in the allotted time with the ‘on’ rate assumed to be ∼2/sec [11], [12], [13]), we use one of two initial conditions. If we want to find the time it takes for a cargo with a single motor to attach, then the cargo is allowed to rotate consistent with Brownian diffusion, and the procedure is repeated. Eventually, the motor binds. The time between when the simulation is started and when the motor attaches is the ‘on’ rate for the cargo; since only one motor is present, it reflects how the presence of the cargo affects the motors' on-rate.

The other initial condition is used if there are multiple motors and we are more interested in transport along the microtubule after the motors attach to the filament. In this case, if none of the motors attaches after being given the opportunity to do so, the cargo is rotated so that at least one motor attaches to the microtubule.

Once some subset of the motors is attached, the cargo travels along the microtubule. At each time step of the simulation, each motor on the cargo is given the opportunity to detach from the MT if it is attached, or attach if it is detached (and geometrically can reach the MT). If a motor is attached to a MT, then there is some probability that it will bind and hydrolyze ATP, and subsequently take a step. Although kinesin is a two headed motor, we model each motor by a single kinesin head that hydrolyzes ATP in such a way that Michaelis-Menten kinetics is obeyed. The probabilities of a motor detaching from the MT, releasing ATP, and taking a step are all dependent on the load on the cargo because the cargo exerts force on the motors (see Text S1. This load has contributions from the externally applied force, the other motors which are pulling the cargo, and from thermal fluctuations. The thermal fluctuations randomly rotate and translate the cargo which, in turn, can stretch the motor linkage and exert a load on the motor. (See below for further details on thermal fluctuations.) Once all the motors have been given a chance to step, the cargo is translated and rotated according to the force and torque to which it is subjected. The cargo travels along the microtubule until all the motors detach from the microtubule, and the ‘run’ ends; this then determines the run length of the cargo. The velocity is calculated by dividing the distance the cargo moves by the travel time τ, where τ is typically 1 msec but may be as long as 10 msec. Averaging over these velocities gives the average velocity. To get good statistics, we simulate a specified number of runs with the same initial conditions to get a set of runs. We also simulate a number of sets with different initial conditions to obtain good statistics.

In our simulations, the spherical cargo is subjected to thermal fluctuations which we can divide into translational and rotational components. The equation of the cargo's translational motion is given by the Langevin equation:
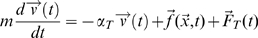
(1.1)where *m* is the cargo's mass and 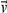 is the cargo's velocity. The drag force on the cargo is proportional to its velocity with the drag coefficient 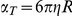, where *R* is the cargo's radius and 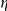 is the coefficient of viscosity which is the kinematic viscosity multiplied by the specific gravity of the fluid. 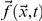 is the sum of the forces due to an external force of magnitude F_L_ and the force of the engaged motors pulling on the cargo. We solve this equation in the Text S1, and quote the solution here for the position of the cargo at time step *t+Δt*:
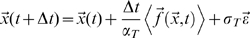
(1.2)where 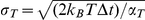 is the standard deviation of a normal distribution and 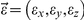 is a vector in Cartesian coordinates of the laboratory frame of reference that represents three independent random variates drawn on a normal distribution having zero mean and unit standard deviation.

For the cargo's rotational motion, the corresponding Langevin equation is
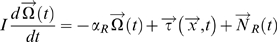
(1.3)where 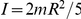 is the moment of inertia of a solid spherical cargo, and 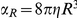 is the drag coefficient proportional to the angular velocity 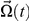. 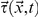 is the torque on the cargo referenced from the center of mass due to the engaged motors. 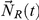 is the rapidly varying random torque due to the thermal fluctuations of the environment. We solve this equation in the Text S1 where we give the formulas for the change in orientation of the cargo at each time step. These formulas are analogous to Eq. (1.2). As we shall see, rotational diffusion due to thermal fluctuations can play a significant role in limiting the distance that a cargo can travel.

**Figure 2:**
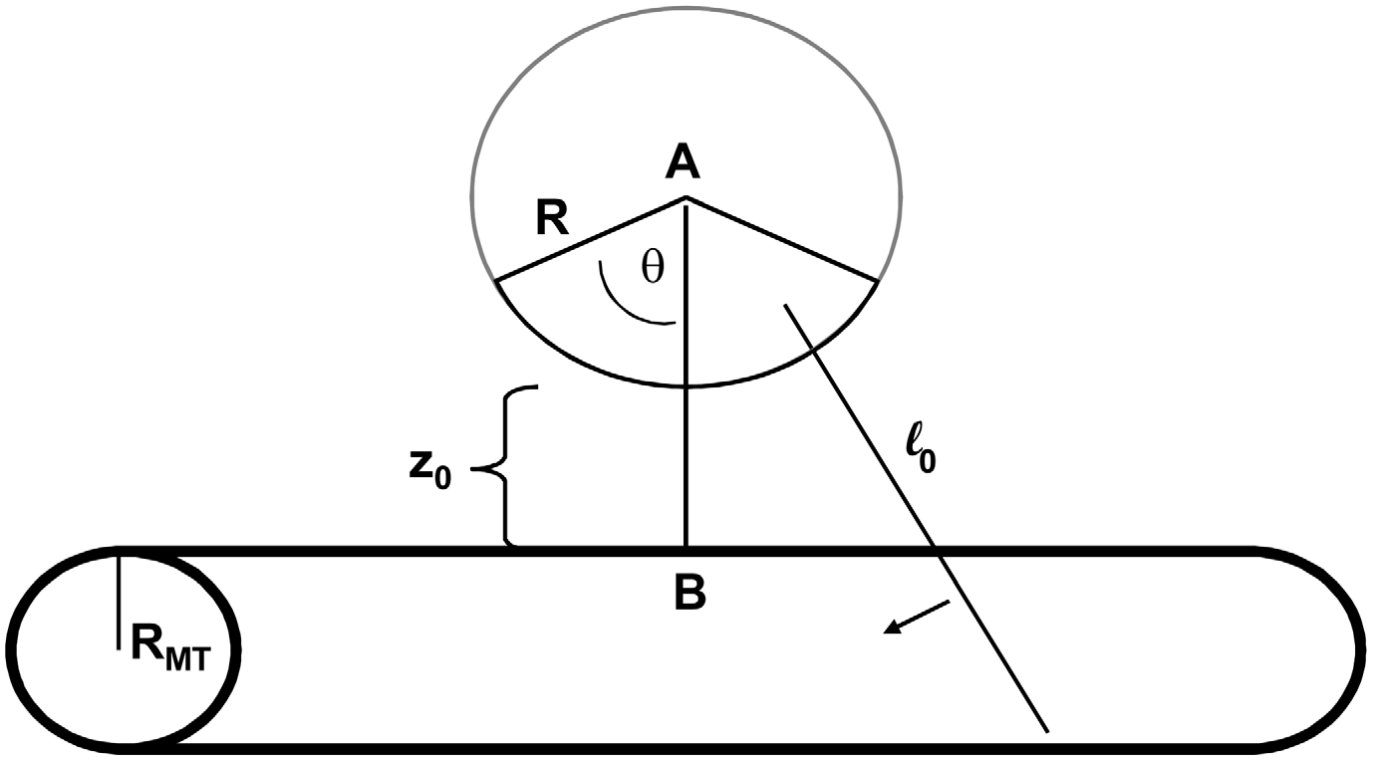
Cluster angle.

After considering motors randomly attached anywhere on the cargo, we consider cases which have a restricted region of the cargo surface area where motors can attach. For these cases, we start each simulation with N motors randomly attached to the cargo's surface within a region specified by the cone angle as shown in Figure 2. The area available for attachment can be described by a cone with its apex at the center of the sphere. A line extends from the apex to the base of the cone. The cluster angle φ is the angle between this line and the side of the cone. The intersection of the cone with the surface of the cargo defines the allowed region of motor attachment. The cluster angle can vary between 0 and 180 degrees. A cluster angle of 90 degrees defines the lower hemisphere of the cargo. A cluster angle of 180 degrees corresponds to the entire spherical surface, and means that the motors can attach anywhere on the sphere.

## Results

We organize our results according to the questions posed in the introduction.

### A. How does a cargo affect the rate at which the motor(s) on the cargo bind to the microtubule?

### A. i. For a single motor on a cargo, how does the presence of the cargo affect the motor's effective ‘on’ rate, i.e., the rate at which the motor binds to the microtubule?

#### The cargo affected a motor's ‘on’ rate

**Figure 3:**
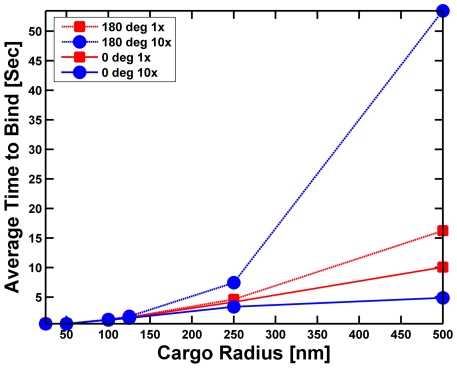
The average time for a motor (with an on-rate of 2/sec) to bind to a microtubule. The motor is initially attached to either the South Pole (cone angle = 0, solid lines) of the cargo facing the microtubule or randomly attached somewhere on the cargo (cone angle = 180 degrees, dashed lines). The cargo rests on the microtubule, and rotates randomly due to thermal effects but cannot diffuse away from the microtubule. The simulations were run long enough to allow at least 95% of the cargos to bind to the microtubules. The binding time was the averaged over the cargos that bound to the microtubule in this time. 1× represents a viscosity equal to that of water and 10× means the viscosity is 10 times greater than that of water. We took the viscosity of water to be 10^−9^ pN-s/nm^2^ throughout the paper.

Let us start with our investigation of the effect of the presence of the cargo on motor attachment to a microtubule. Kinesin is estimated to have an on-rate of approximately 2 sec^−1^, so that one expects a free motor close to a microtubule to take roughly 0.5 seconds to attach to the microtubule. If we attached that same motor to a small (25 or 50 nm radius) cargo, and held the cargo on the microtubule so that it could rotate randomly due to thermal motion but not diffuse away, we saw that the presence of the cargo had little effect on the typical time for the motor to bind (Figure 3). However, larger cargos started to have a significant effect on the time for a motor randomly positioned on the cargo to attach to the microtubule. Indeed, for a cargo with a radius of 250 nm, the typical time to attach increased to more than 4 seconds, and for a 500 nm cargo the time is up to 16 seconds. As might be expected, because viscosity slows rotational diffusion, increasing the viscosity significantly increased the typical binding time for motors attached to large beads—for a viscosity 10 times that of water, for a 500 nm cargo the typical attachment time is up to 53 seconds. Thus, we concluded that the presence of the cargo can indeed have a very significant effect on the typical time for a motor attached to the cargo to bind to a microtubule.

### A. ii. How does the cargo's size and viscosity affect the probability that the cargo will bind to the microtubule before diffusing away?

#### The cargo affected the percentage of ‘free’ cargos that bound to the microtubule

**Figure 4:**
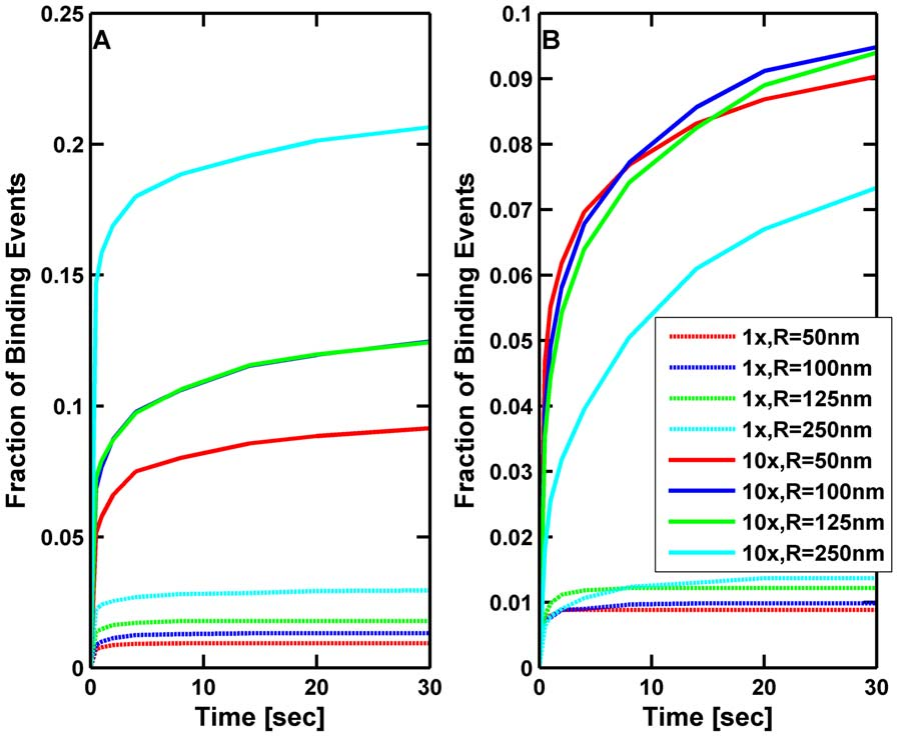
Fraction of cargos with a single motor that have bound to the microtubule versus time in an environment with the viscosity of water (1×) and 10 times the viscosity of water (10×). The cargos are initially resting on the microtubule, and can rotate and diffuse away due to thermal fluctuations. In the first plot (A), the simulation was started with the motor located at the South Pole facing the microtubule, and in the second plot (B), the motor was randomly placed anywhere on the cargo. Each curve represents the outcome of 6000 trials.

In Figure 4 we plot the fraction of cargos with a single motor that bind to the microtubule as a function of the time allowed for different cargo sizes and different viscosities. In contrast to the study in Figure 3 where cargos were not allowed to diffuse away from the microtubule, in this study the cargos started by resting on the microtubule, and then could both rotate and diffuse away as a result of thermal fluctuations. A cargo was given up to 30 seconds for its motor to bind. If it had not bound in that time or if it diffused a distance greater than 50 times the length of the motor (5500 nm), then the trial was deemed a failure. The fraction of successful binding events as a function of time is plotted in Figure 4. When the motor was closest to the microtubule, the larger the cargo and the larger the viscosity, the greater was the probability of binding because these were the cases where there was the least amount of rotational and translational diffusion. When the motor was randomly attached somewhere on the cargo, larger cargos still had the greatest chance of binding at low viscosities and long times. However, for large viscosity, having the motor randomly attached to the cargo reduced its chances of binding since, if the motor could not reach the microtubule, it had a harder time to come within reach before the cargo diffused away from the microtubule. Nonetheless, it is clear overall that higher viscosity improves the cargo's binding rate—rotational diffusion tends to bring the motor close to the MT before the cargo diffuses too far from the MT, so the presence of the cargo dramatically increases the fraction of successful binding events at high viscosity.

### A. iii. Further, how does the length of the motor compared to the cargo size contribute to these properties?

#### The motor's length was an important contributor to its effective on-rate

**Figure 5:**
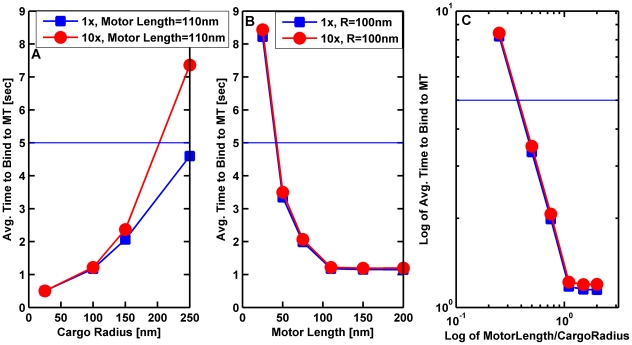
Average time for a cargo with a single motor to bind to a microtubule as a function of cargo radius and motor length. (A) Average time for a 110 nm motor to bind to a microtubule versus cargo radius. (B) Average time for a motor bound to a cargo with radius 100 nm to bind to a microtubule as a function of motor length. (C) Log-log plot of the average time to bind (in seconds) versus the ratio of motor length to the cargo radius. The solid horizontal line marks a time to bind of 5 seconds. The cargo was initially resting on the MT with the motor randomly placed on the cargo's surface. The cargo was allowed to rotate randomly due to thermal effects, but it could not diffuse away from the microtubule. The simulations were run long enough to allow at least 95% of the cargos to bind to the microtubules. The binding time was the averaged over the cargos that bound to the microtubule in this time. The solid lines are guides to the eye. In all 3 plots the blue squares correspond to the viscosity of water (1×), and the red circles correspond to 10 times the viscosity of water.

When considering the system of the motor plus cargo, one might wonder how important the physical length of the motor is. We investigated this in the context of the on-rate. We varied the cargo radius from 25 to 250 nm, the motor length from 25 to 1000 nm, and the viscosity from that of water to 10 times that of water. As might be expected, the higher the viscosity, the longer it took for a single motor attached to a cargo to bind to a microtubule. In addition, motors with shorter stalks on average tended to take longer to bind to the microtubule. Interestingly, we discovered that the effective on-rate improved until the motor was approximately equal to the cargo's radius; for motors much longer than the cargo's radius there was little additional improvement (Figure 5). Biologically, the relevant time to bind should be at most a few seconds. Again we found that in order to have the time to bind to a microtubule be less than or equal to 5 seconds, the motor needed to be longer or comparable to the radius of the cargo. In order to maximize the on-rate for a cargo driven by one or two motors, our model thus suggests that one might therefore choose to have the motors be comparable to the cargo's radius. Axonal cargos driven by kinesin are rarely larger than 200 nm in diameter [14], [15], [16]; consistent with this, kinesin is 110 nm long. Below, we investigate this question more completely, with multiple motors.

#### A cargo moved by a single motor had unexpected oscillations

**Figure 6:**
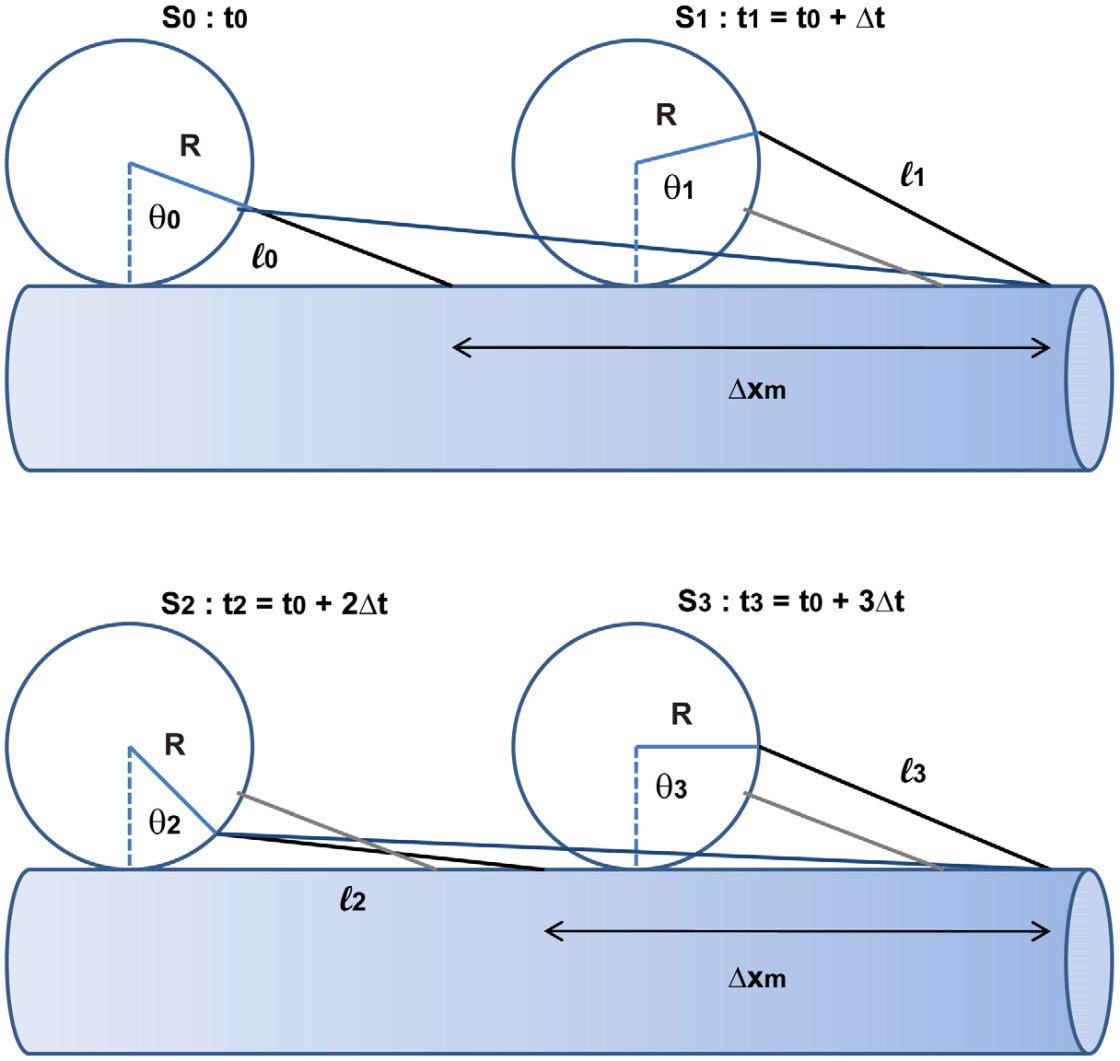
First few steps of a cargo pulled along a microtubule by a single motor. The figure illustrates porpoise-like oscillations about the equilibrium point.

**Figure 7:**
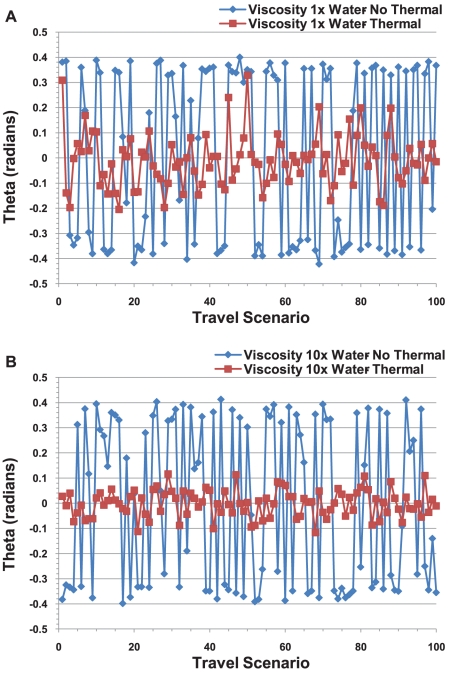
Rotation angle θ at which the motor detached during each of 100 Monte Carlo simulations of a cargo with 500 nm radius pulled by a single motor in a medium with the viscosity of water and 10 times the viscosity of water. The cluster angle is zero. Blue lines, labeled “no thermal,” correspond to the case of no thermal rotational and no thermal translational diffusion. Red lines, labeled “thermal,” correspond to the presence of both thermal rotational and thermal translational diffusion.

When we considered a cargo hauled along a microtubule by a single active motor without any thermal fluctuations, we found that the motor and cargo underwent an oscillating porpoise-like motion as the cargo traveled down the microtubule. This is illustrated in Figure 6. The upward and downward angular motion produced a load on the motor by tugging on the motor linkage. The oscillations, and hence the tugging, became more pronounced as the cargo radius increased. These oscillations were damped by viscosity and thermal effects as seen in Figure 7 where we show the oscillations in the angle θ (measured with respect to the z-axis perpendicular to the microtubule) at which the cargo's motor detached from the microtubule in the different simulation runs. This implies that viscous damping in the cell will render these oscillations of no consequence.

### A. iv. What about the binding probability of a cargo with multiple motors?

#### How multiple motors functioned together to haul a cargo

**Figure 8:**
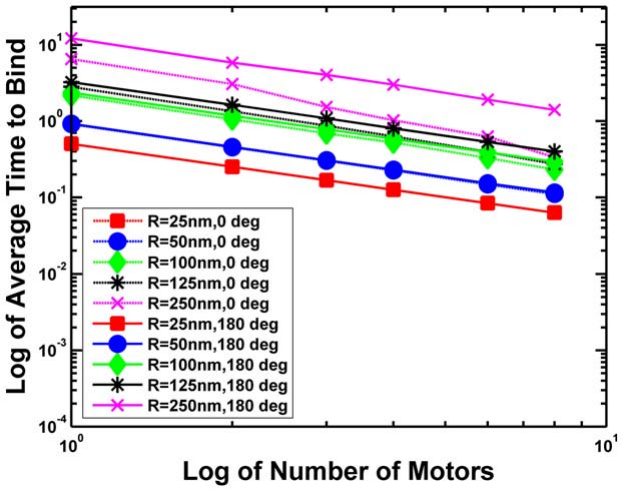
Log-log plot of the average binding time versus the number of motors on a cargo placed 50 nm above a microtubule. The different lines correspond to different cargo radii ranging from 25 nm to 250 nm. The cargo is allowed to rotate due to thermal diffusion but it cannot diffuse away. The medium has the viscosity of water. Having a cone angle of 180 degrees means that the motors are randomly placed on the cargo's surface. Having a cone angle of 0 degrees means that the motors are all located at the South Pole facing the microtubule.

Since our single motor simulations showed that the presence of a cargo can affect the on-rate of a motor, we wanted to see how the time it took for at least one motor to bind was affected by the total number of motors attached to the cargo as well as the size of the cargo. We considered the case of multiple motors randomly attached anywhere on the cargo surface. We placed motors randomly on the surface of a cargo, and measured the average time that it took to have at least one motor bind to the microtubule. An example of our results is shown in Figure 8. The binding time increases with cargo radius, and decreases as 1/N where N is the total number of motors on the cargo.

#### Dependence of cargo binding time on motor length and the number of motors

**Figure 9:**
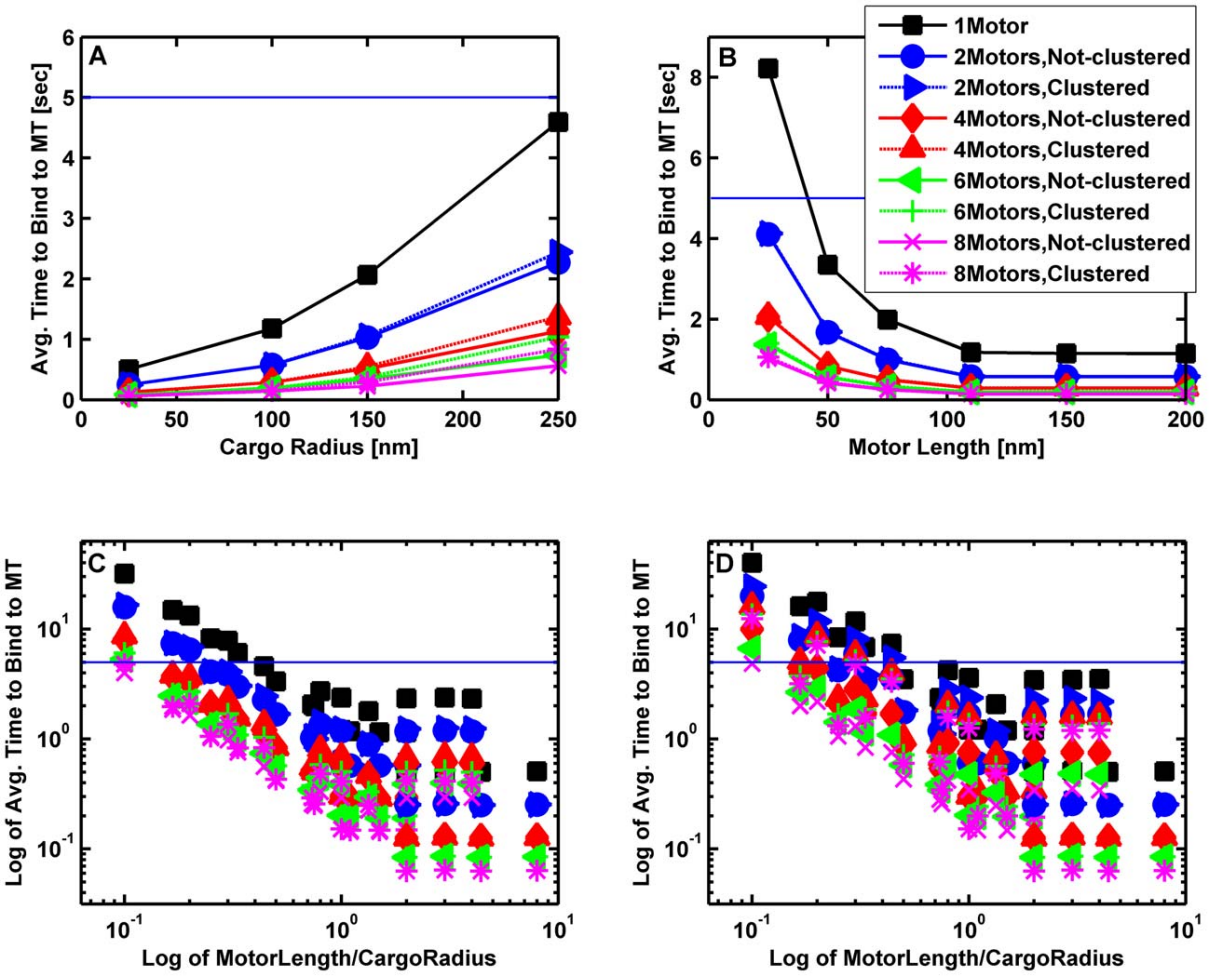
Average time for a cargo with up to 8 motors to bind to a microtubule as a function of cargo radius and motor length. (A) Average time for 110 nm motors to bind to a microtubule versus cargo radius at the viscosity of water. (B) Average time for motors bound to a cargo with radius 100 nm to bind to a microtubule as a function of motor length at the viscosity of water. (C) Log-log plot of the average time to bind (in seconds) versus the ratio of motor length to the cargo radius at the viscosity of water. (D) Log-log plot of the average time to bind (in seconds) versus the ratio of motor length to the cargo radius at 10 times the viscosity of water. The solid horizontal line marks a time to bind of 5 seconds. The motors are either clustered at one point on the surface of the cargo or they are randomly distributed over the surface of the cargo. The cargo was allowed to rotate randomly due to thermal effects, but it could not diffuse away from the microtubule. The log-log plots show that the time to bind goes as L^−b^ where L is the length of the motor and the exponent b varies between 1.3 and 1.7. The solid and dashed lines are guides to the eye.

Above, we investigated how the motor's length affected on-rate in the single-motor case. We extended these studies to determine the time it took for a motor to bind to a microtubule, as a function of the motor length (25 to 1000 nm), the cargo radius (25 to 250 nm), and the number of motors on the cargo. For these simulations the cargo rested on the microtubule and was not allowed to diffuse away, but did rotate randomly due to thermal effects, so that if the motor(s) were initially unable to reach the cargo, they soon came within reach of the microtubule. The motors were either clustered at one random point on the cargo's surface or else they were spread randomly over the surface. We tried motors of different lengths and cargos of different sizes. Our results are shown in Figure 9. We found that the average time τ_bind_ that it took for the first motor to bind to the microtubule had a power law dependence on the length *L* of the motor when the motor was shorter than or comparable to the cargo radius; namely, τ_bind_∼L^−b^ where the exponent b varied between 1.3 and 1.7. This meant that the longer the motor, the lower the average binding time. When the motor was much longer than the cargo radius, the average binding time became independent of the motor length. For motors short compared to the radius of the cargo, the binding time decreased as the number of motors increased. But as the motor length increased, the time it took to bind was less sensitive to the number of motors on the cargo. We can see that from Figure 9 that the binding time for kinesin, which is 110 nm long, is rather insensitive to the number of motors on the cargo. This may be one reason why molecular motors involved in intracellular transport are not shorter than about 60 nm which is the length of dynein [17]. If motors involved in transport were shorter, it would take too long for cargos to attach to filaments unless there were a large number of motors on the cargo. Intriguingly, some have suggested that more dyneins may be functioning on a cargo than kinesins [3], [4]. While this remains to be fully established, from an on-rate point of view, additional dynein motors present could compensate for dynein's shorter overall length relative to kinesin.

### A. v. How does the distance between microtubules affect the probability that a cargo with one or more motors will bind to a microtubule?

#### Rapidly diffusing cargos can easily reach and bind to nearby microtubules

So far we have only considered how long it takes a cargo that is sitting on a microtubule to actually bind to a single microtubule, but unattached cargos may not start on a microtubule. Rather they may be floating in the cytoplasm and may need to come within reach of a microtubule. In this case the time for a motor on a cargo to bind to a microtubule depends on the time to diffuse to a microtubule, and once it finds a microtubule, to bind to it before the cargo diffuses away. The time to diffuse between microtubules depends on the viscosity, the size of the cargo, and the distance between microtubules. The typical intracellular environment consists of multiple microtubules extending radially from the nucleus. In a flat 2D cell, the microtubule density decreases as 1/r where r is the distance from the microtubule organizing center (MTOC). At the periphery of *Xenopus* melanophores, the typical microtubule separation is about 800 nm [18].

We performed simulations to see how the binding fraction (the fraction of cargos where at least one motor binds to a microtubule) depends on the distance between microtubules, the radius of the cargo, and the viscosity. The geometry was a slab (extending from z = −1 micron to z = +1 micron) with either one microtubule or an infinite number of evenly spaced microtubules running parallel to the y-axis. For the case of one microtubule, it lays along the y-axis with the plus end in the positive y-direction, and slab extended to infinity in the x and y directions. For the case of microtubules evenly spaced by a distance x_MT_, we placed one along the y-axis, and the next one a distance x_MT_ away. To obtain an infinite number of microtubules, we used periodic boundary conditions in the x direction such that if a cargo has a position x > x_MT_ (x < x_MT_), we mapped x to x−x_MT_ (x+x_MT_). Initially the cargo rested on a microtubule with motor(s) attached randomly on the surface of the cargo. The cargo was allowed 60 seconds to attach; if it failed to attach in that time, the trial was deemed a failure. The cargo was able to diffuse translationally and rotationally. The z-position of cargos that entered the floor or ceiling in the z direction was reset such that the cargo just touched the floor or ceiling. We considered microtubule spacings between 400 and 1200 nm, cargo radii from 25 nm to 250 nm, and viscosities from that of water to 1000 times that of water. We ran 1000 trials for a given set of values of the parameters.

**Figure 10:**
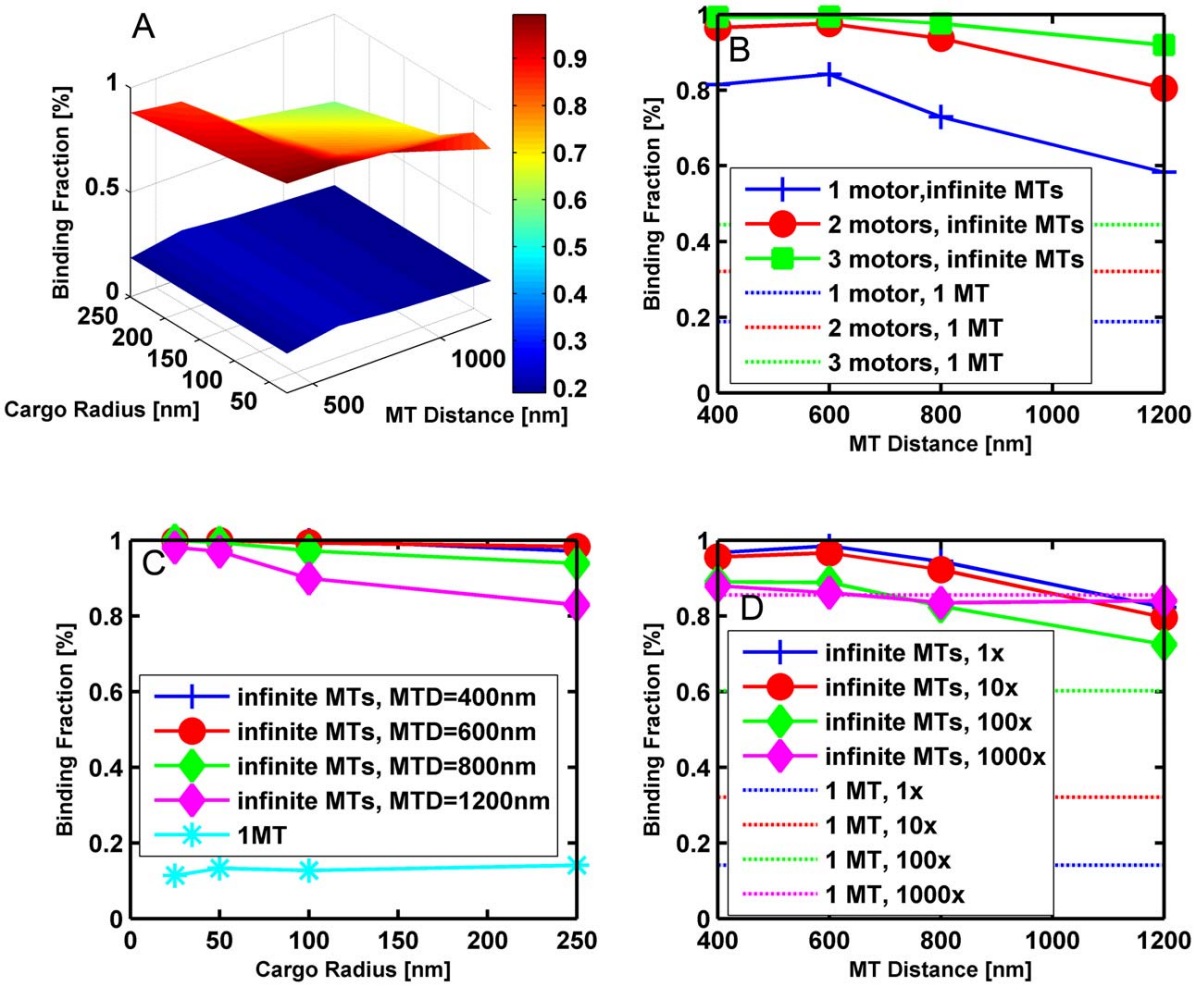
Binding fraction (fraction of cargos that bind to the microtubule in 60 seconds) vs. cargo radius and the distance between microtubules. (A) 3D plot of binding fraction vs. cargo radius and microtubule (MT) distance for a cargo with 1 motor randomly attached on its surface at 10 times the viscosity of water for both translational and rotational diffusion. The lower blue surface is for the case of 1 microtubule where there is obviously no dependence on microtubule distance. The upper surface is for the case of an infinite number of microtubules. (B) Binding fraction vs. microtubule distance for a cargo with radius 250 nm at 10 times the viscosity of water for both translational and rotational diffusion. The solid lines are for an infinite number of microtubules, while the single microtubule case is represented by the straight dot-dash lines which have no dependence on microtubule distance. Blue pluses, red circles, and green squares are for 1, 2, and 3 motors, respectively, placed randomly on the surface of the cargo. (C) Binding fraction vs. cargo radius for a cargo with 2 motors randomly attached to its surface at the viscosity of water for translational diffusion and 10 times the viscosity of water for rotational diffusion. MTD stands for microtubule distance, i.e., the spacing between microtubules. (D) Binding fraction vs. microtubule (MT) distance for a cargo with a radius of 250 nm and 2 motors randomly attached to its surface at 10 times the viscosity of water for rotational diffusion. The solid lines are for the case of an infinite number of microtubules and the dot-dash lines are for one microtubule. Blue, red, green, and magenta correspond to 1, 10, 100, and 1000 times the viscosity of water for translational diffusion.

Our results for the binding fraction are shown in Figure 10. One can see that the binding fraction was much higher when an infinite number of microtubules were available compared to just one microtubule for viscosities up to 100 times that of water. This was because the time to diffuse between neighboring microtubules was relatively short. So if a cargo drifted away from a microtubule, it quickly found another one. However, if only one microtubule was available, the cargo tended to diffuse away before it had a chance to bind. When the viscosity was 1000 times that of water, the cargo was so immobile that it was localized near its initial microtubule and did not visit other microtubules. As a result, the binding fraction for an infinite number of microtubules was about the same as for 1 microtubule as seen in Figure 10D. As expected for the case with multiple microtubules, the binding fraction decreased somewhat with increasing distance between the microtubules (see Figure 10B and 10C), with increasing cargo size (see Figure 10C), and with increasing viscosity (see Figure 10D).

### B. Effect of thermal fluctuations on cargo travel distance

### B. i. Does the cargo's Brownian motion affect the motor's function as measured by its travel distance for a cargo with a single motor?

#### Run length decreased with increasing viscosity at large cargo sizes, due to diffusive rotation as well as viscous drag

**Figure 11:**
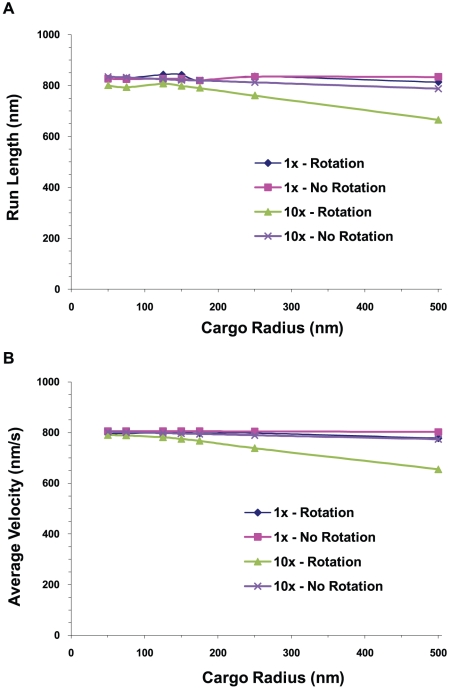
Average run length and average velocity of a cargo vs. cargo radius. (A) Cargo run length and (B) cargo velocity vs. cargo radius for a cargo hauled by a single motor with and without rotation at the viscosity of water and 10 times the viscosity of water.

The presence of the cargo could also affect motor function by amplifying thermal noise effects, leading to increased random forces acting on the motor, and thus possibly affecting travel distance. We considered a cargo being hauled along a microtubule by a single active motor. The radius of the cargo ranged from 50 nm to 500 nm, and we studied environments with the viscosity of water and 10 times the viscosity of water. In Figure 11 we show the results where we plot the average cargo run length and velocity versus the cargo radius with and without rotational diffusion for different viscosities. Translational diffusion was present in all cases, and the run length was the distance traveled by the cargo before falling off the microtubule. Notice that the run length and velocity were approximately independent of cargo size when both rotational and translational diffusion were present for low viscosity. However, at 10 times the viscosity of water, when there was rotational diffusion, the run length and velocity decreased by about 20% for large cargo sizes, implying that rotational diffusion can play a significant role in decreasing run lengths under certain conditions. Looking at Equation (1.3), we see that the reason is related to the increased drag torque 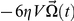 which increases with viscosity *η* and cargo volume *V*. This increased drag torque reduced the rotations at each time step, but when there was a large random rotation that stretched the motor linkage, it lasted longer, increasing the time that there was load on the motor and the probability of detachment.

**Figure 12:**
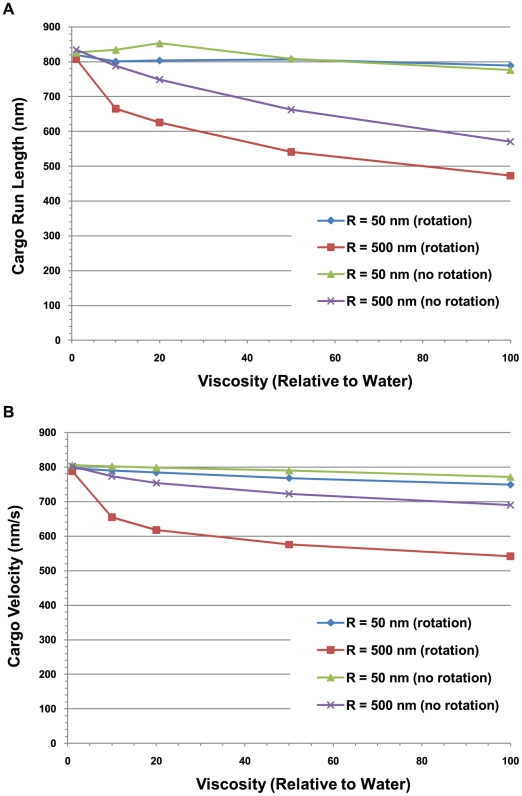
Average cargo run length and velocity vs. viscosity. (A) Run length and (B) average velocity of a cargo carried by a single motor vs. the viscosity relative to water for 50 nm and 500 nm radius cargos both with and without rotational diffusion.

The contribution of rotational diffusion in limiting the run length continued out to higher viscosities, though not quite so strikingly. This is seen in Figure 12a where we show the run length versus viscosity for small (R = 50 nm) and large (R = 500 nm) cargos hauled by a single motor both in the presence and absence of rotational diffusion. Here we see that increasing the viscosity to 100 times that of water had little effect on the run length of the small cargo, but resulted in a decrease of the run length of the large cargo by about 40% when rotational diffusion was present and by about 30% when there was no rotational diffusion compared to the case when the viscosity was that of water.

Figures 11b and 12b show that viscosity can have a significant effect on the average velocity of large cargos. There was little effect of viscosity on the velocity of small cargos, but when the viscosity was increased by a factor of 100, the velocity of large cargos dropped by about 30% when rotational diffusion was present but only by about 14% when there was no rotational diffusion.

### B. ii. Does the cargo's Brownian motion affect the motor's function as measured by its travel distance for a cargo with multiple motors?

#### Thermal motion that increased with cargo size decreased run lengths

As we discussed for the case of one motor on a cargo, a cargo that was tethered to a filament by one or more motors was subjected to thermal motion in the form of translational and rotational diffusion. These thermally generated forces and torques increased with the size of the cargo according to Stokes' law which says that the translational drag coefficient α_T_ = 6πηR, and the rotational drag coefficient α_R_ = 8πηR^3^. As a result, the run lengths decreased with increasing viscosity and cargo radius for a fixed number of motors and cluster angle. We saw this for the case of one motor (Figures 8 and 9) where the run length decreased noticeably at large cargo sizes at the larger viscosity due to the increases in drag and thermal fluctuations.

**Figure 13:**
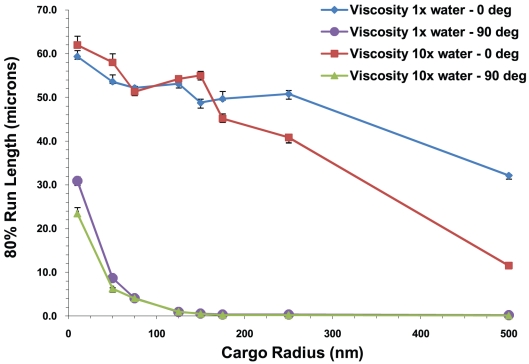
20^th^ percentile run length vs. cargo radius for 5 motors at the viscosity of water and 10 times the viscosity of water for cluster angles of 0 and 90°. The error bars underestimate the error and the curves would be made smoother by increasing the sample space. The roughness of the curves is not indicative of any physical behavior.

We show the case of multiple motors in Figure 13 where the 20^th^ percentile run lengths L_80_ are plotted versus cargo radius for 5 motors at different values of the viscosity and cluster angle. By 20^th^ percentile run length, we mean the run length such that 80% of the motors traveled at least this far. We denote this run length by L_80_. One can clearly see that L_80_ decreased with increasing cargo radius. This decrease was accentuated by increasing viscosity, especially in the simplest case of zero cluster angle.

To further investigate the importance of rotational diffusion, we measured L_80_ for 5 motors for a sphere with R = 500 nm at the viscosity of water, and R = 250 nm at twice the viscosity of water. Note that the translational drag coefficient α_T_ was the same for both cases, but α_R_ was larger for the 500 nm sphere. We found that L_80_ was the same in both cases if we turned off rotational diffusion, but if we included rotational diffusion, then L_80_ (R = 250 nm) = 9.9 microns compared to L_80_ (R = 500 nm) = 7.7 microns. Clearly rotational diffusion of the cargo played an important role in increasing the load on the motors and decreasing the travel distance.

### C. Number of engaged motors

### C. i. For randomly distributed motors, does doubling, say, the total number of motors on the cargo double the number of motors engaged in hauling the cargo along the microtubule?

#### The number of engaged motors depended on the total number of motors randomly placed on the cargo and the cargo's size

**Figure 14:**
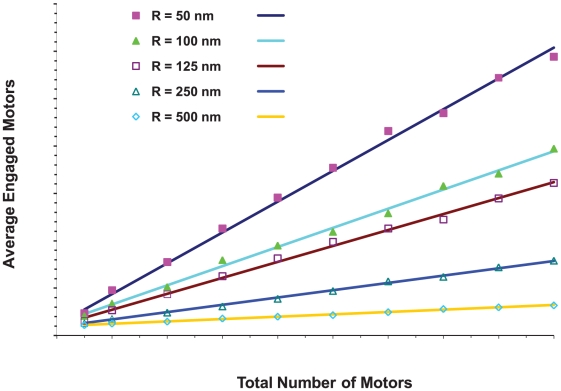
Average number of engaged motors vs. total number of motors at the viscosity of water for various cargo radii. Plot at 10× viscosity of water is similar.

Since our simulations suggested that the presence of the cargo can affect both effective motor ‘on’ rates and the mean travel distance, we were also interested in how multiple motors attached to the cargo might function. We first considered motors randomly on the surface of a cargo, and during the simulation, calculated the average number of engaged motors as well as the distribution of the number of engaged motors as a function of the total number N of motors on the cargo. This was done as a function of the cargo radius R, for both the case where the cargo moved through a medium with the viscosity of water, and also for a medium with10 times the viscosity of water. The results in Figure 14 show that the average number of engaged motors increased linearly with the total number N of motors on the cargo of a fixed radius R. However, the slope of the line decreased significantly as the radius of the cargo increased, and for fixed N, the average number of engaged motors decreased quite rapidly with increasing R, because the active motors that can carry the cargo along a filament must be able to reach the filament. Thus, if motors are randomly distributed over the surface of the cargo (corresponding to a cluster angle of 180 degrees as described above), the number of motors that can reach the cargo depends on the diameter of the cargo compared to the length of the motor.

**Figure 15:**
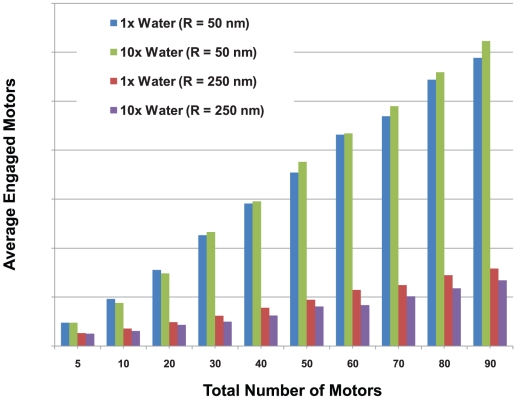
Average number of engaged motors vs. total number of motors for cargos with radii of 50 nm and 250 nm in media with a viscosity equal to that of water and 10 times that of water. The number of engaged motors grows more slowly for larger cargos.

From a biological point of view, the relationship between the number of motors engaged and the total number of motors present suggested that for most cargo sizes, doubling the total number of motors on the cargo did not double the number of engaged motors. Put differently, even though the number of actively engaged motors is linearly proportional to the total number N of motors on the cargo, it does not necessarily follow that the number of engaged motors will double if the total number of active motors on the cargo doubles because the constant of proportionality may be too small to correspond to doubling the number of active motors (Figure 15). (By active motors, we mean the number of motors that are able to haul the cargo even though they may not actually be doing so. In other words active motors have not been inactivated or incapacitated by some interfering protein or some conformational change.) For example, consider a cargo with a 100 nm diameter (R = 50 nm). If N = 5, the average number of engaged motors was 2.4. If the total number of motors is 10 times larger (N = 50), the average number of engaged motors was 17.7 or approximately 6 times larger. In contrast, for a 500 nm diameter cargo (R = 250 nm), if N = 5, the average number of engaged motors was 1.3. But with10 times as many motors (N = 50), the average number of engaged motors was merely tripled to 4.7.

What this means from a regulatory point of view is that, in the absence of some higher-order organization of motors (see below), to control motion by recruitment of motors to the cargo, different numbers of motors must be recruited depending on the cargo size. In the two cases above, if the number n of engaged motors is approximately 1, to recruit enough motors to end up with n∼2 requires one to quadruple the total number of motors present in the R = 50 nm case, but increase the total number of motors by a factor of 20 in the R = 250 nm case.

Alternatively, one could hypothesize that motor recruitment could be controlled locally, so that the surface density of motors could be controlled. Interestingly, because of geometrical effects, fixed densities of motors did not equate to the same number of engaged motors on cargos of different sizes. For example, a density of 6.3×10^−5^ motors/nm^2^ corresponded to 2 total motors (and 1.5 engaged motors) on a cargo with R = 50 nm, but to 50 total motors (and 4.7 engaged motors) on a cargo with R = 250 nm. The relationship between motor density and the number of engaged motors is investigated more fully below.

#### Use of Poisson statistics to estimate the number of engaged motors

**Figure 16:**
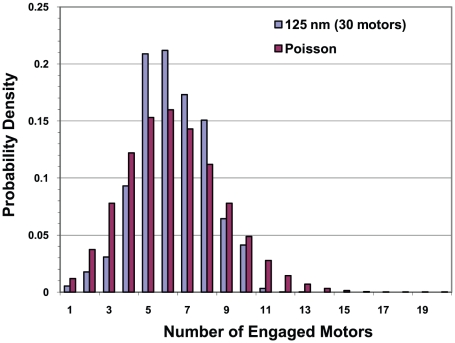
Distribution of the number of engaged motors at the viscosity of water. 30 total motors on a cargo with radius of 125 nm.

Frequently, one uses Poisson statistics to estimate the number of motors that are engaged. We were able to directly test this assumption. By recording the number of engaged motors at each time step, we were able to calculate the distribution P(N_engaged_) of engaged motors. We found that P(N_engaged_) obeyed Poisson statistics to a good approximation that improved as the total number N of motors increased. For 30 motors or more, it was an excellent approximation. An example is shown in Figure 16. For a given total number of motors on the cargo, the only adjustable parameter is the Poisson mean for which we used the simulation results to obtain average number of engaged motors.

### C. ii. What is the relationship between the cargo size, the viscosity, the number of motors present, the average number of motors actively engaged in transporting the cargo, and the cargos' mean travel distance?

#### Relationship between the cargo size, the motor density, and the number of engaged motors

**Figure 17:**
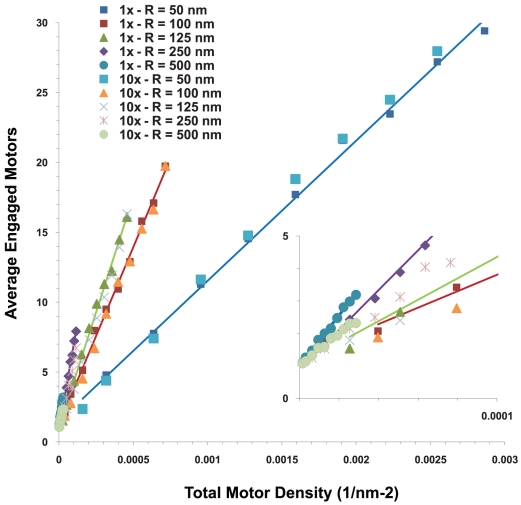
Average number of engaged motors as a function of the density of motors on the cargo surface for a variety of cargo radii. The lines are interpolated between points with the viscosity of water. The points at 10 times the viscosity of water have no lines. Inset is a blow-up of the region near the origin.

If motors were randomly distributed on a cargo, a key quantity that helped to determine the number of engaged motors, and hence the run length, was the density of motors on the cargo surface. Figure 17 shows the average number of engaged motors as a function of the surface density of motors. In general, the larger the cargo, the lower the motor density required to achieve a specific mean number of engaged motors.

In our example above, 5 motors on a cargo with a diameter of 100 nm correspond to a density of 1.6×10^−4^/nm^2^, and 50 motors correspond to a density of 1.6×10^−3^/nm^2^. This is in contrast to a 500 nm diameter cargo where density of 6.4×10^−6^/nm^2^ corresponds to 5 motors, and a density of 6.4×10^−5^/nm^2^ corresponds to 50 motors. This is summarized in Table 1.

**Table 1:** Relationship between the cargo size, the motor density, and the number of engaged motors.

Radius [nm]	Total number of Motors	Density [nm^−2^]	Number of Engaged Motors
50	5	1.6×10^−4^	2.4
50	50	1.6×10^−3^	17.7
250	5	6.4×10^−6^	1.3
250	50	6.4×10^−5^	4.7

In addition to motor density, cargo size is also important because motors have a harder time reaching the microtubule from a large cargo. Since the motors cannot pass through the cargo, there is more excluded volume for large cargos.

### Non-random organization of multiple motors on cargos

### D. For motors “clustered” on the cargo, what is the relationship between cargo size, the viscosity, the size of the cluster, the number of motors in the cluster, and the mean travel distance?

#### The run length increased exponentially with the total number of motors on the cargo

**Figure 18:**
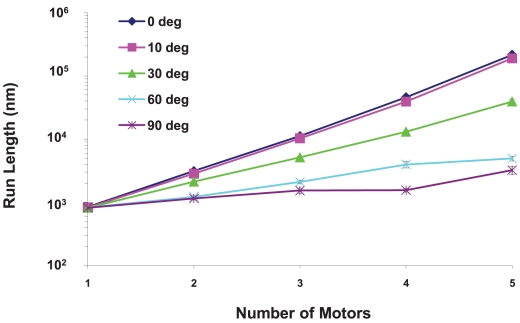
Semi-logarithmic plot of the run length for a 250 nm cargo vs. the total number of motors for different cluster angles at the viscosity of water. The run length increases exponentially with the number of motors. The error bars underestimate the error and the curves would be made smoother by increasing the sample space. The roughness of the curves is not indicative of any physical behavior.

The simplest non-random organization for a group of motors is to cluster them together. We investigated the effects of clustering by specifying the cluster size in terms of the cone angle subtended by the cluster (see Figure 2). We assumed the clusters to be cylindrically symmetric about the axis of the cone. A 0 degree cluster placed all motors at a single point on the cargo; a 90 degree cluster positioned them randomly on the lower hemisphere between −90 and +90 degrees. If only one motor was attached to the cargo, then the run length was about 800 nm, independent of the cargo radius and cluster angle, though run length did decrease slightly under high viscosity conditions for large cargos. For multiple motors, as the number of motors increased, the run length increased exponentially for a fixed cargo radius and cluster angle. We found this to be true for cargo radii of 50, 75, 125, 150, 175, 250 and 500 nm, cluster angles varying from 0 to 90 degrees, and solvent viscosities equal to that of water and ten times that of water. An example is shown in Figure 18. The increase of run length with the total number of motors makes sense since the cargo was able to continue traveling along the filament even if some of the motors detached from the filament. If some motors were detached and some were still attached to the filament, then the detached motors had a chance to reattach to the filament and help carry the cargo.

#### Run length decreased with increasing cluster angle and increasing cargo radius

**Figure 19:**
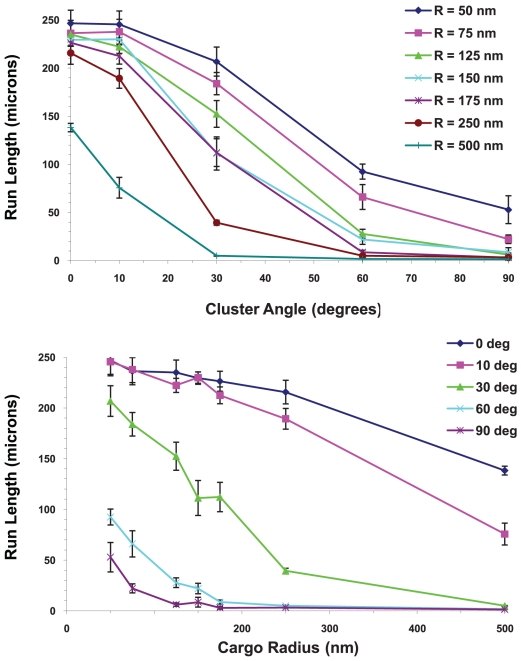
Run length for cargo with 5 motors as a function of cluster angle and cargo radius at the viscosity of water. (A) Run length vs. cluster angle. (B) Run length vs. cargo radius. The error bars underestimate the error and the curves would be made smoother by increasing the sample space. The roughness of the curves is not indicative of any physical behavior.

For two or more motors, the run length decreased as the cluster angle and cargo radius increased. An example is shown in Figure 19 for 5 motors. This decrease in run length was due to the fact that it was harder for the motors to attach to the filament with increasing cargo radius. Increasing the cluster angle increased the cargo surface area where the motors can attach to the cargo; and the farther motors were from the South Pole (nearest point to the filament), the harder it was for them to attach. There were also greater loads on the motors that were barely able to attach, making the probability of detaching from the filament more likely. It is obvious that for larger cargos, clustering is critical to achieve good performance from a limited number of motors.

**Figure 20:**
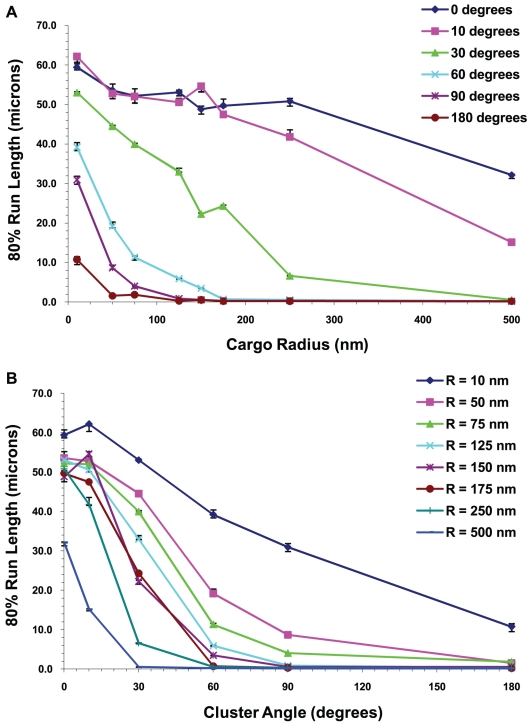
20^th^ percentile run lengths at the viscosity of water. 80% of the cargos traveled at least a distance that we call the 20^th^ percentile run length. 20^th^ percentile run lengths vs. (A) cargo radius and (B) cluster angle for 5 motors. The error bars underestimate the error and the curves would be made smoother by increasing the sample space. The roughness of the curves is not indicative of any physical behavior.

Another way to exhibit this data is shown in Figure 20 where we plot the 20^th^ percentile run lengths L_80_ as a function of the cargo radius and cluster angle. We can see from the figures that L_80_, the minimum distance traveled by 80% of the motors, decreased with increasing cluster angle and cargo radius. This decrease was more rapid for large cargos and for large cluster angles.

#### Run lengths decreased with increasing viscosity

**Figure 21:**
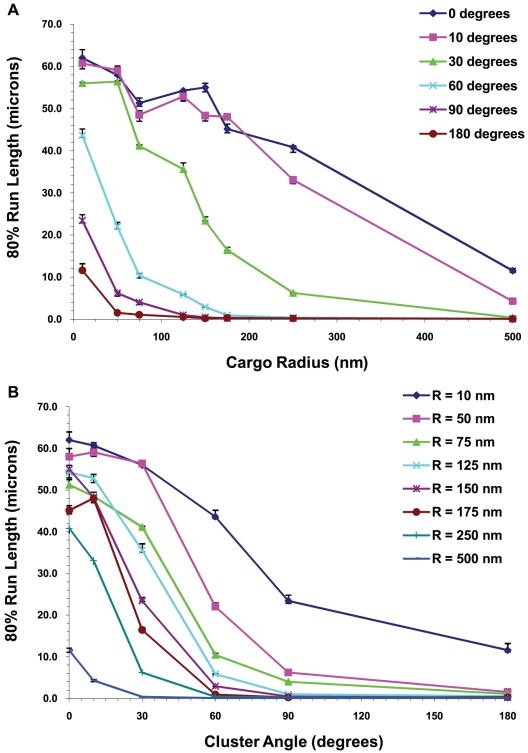
20^th^ percentile run lengths at 10 times the viscosity of water. 20^th^ percentile run lengths vs. (A) cargo radius and (B) cluster angle for 5 motors on the cargo. The error bars underestimate the error and the curves would be made smoother by increasing the sample space. The roughness of the curves is not indicative of any physical behavior.

In Figures 20 and 21, we show the 20^th^ percentile run lengths at the viscosity of water and 10 times the viscosity of water as a function of the cargo radius and the cluster angle. At the higher viscosity, one can see that L_80_ decreased faster with increasing cargo radius and cluster angle. This is to be expected since the higher viscosity medium produced a greater drag on the cargo, and hence a higher load on the motors. In our model the probability of a motor detaching from the microtubule increased exponentially with increasing load [5], leading to shorter run lengths.

#### Surprisingly, clustering motors had little effect on the rate of attaching the cargo to the microtubule

**Figure 22:**
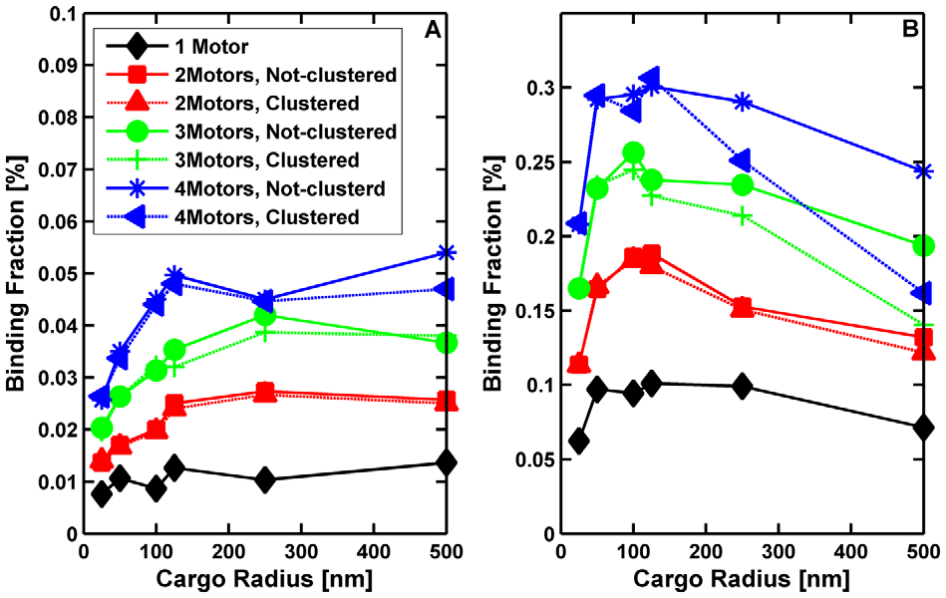
Binding fraction vs. cargo radius for various numbers of motors on the cargo. Each trial was terminated when the cargo bound to the microtubule, or if the cargo did not bind after 60 seconds, or if the cargo did not bind and diffused more than 5.5 microns away from the microtubule. (5.5 microns is 50 times the length of the motor.) The dashed lines correspond to the motors being clustered, i.e., attached to the cargo at one point. The solid lines correspond to the motors being randomly distributed over the surface of the cargo. The cargo initially was resting on the microtubule, and was allowed to randomly rotate and diffuse away due to thermal fluctuations. (A) Medium had the viscosity of water. (B) Medium had ten times the viscosity of water. The curves would be made smoother by increasing the sample space. The roughness of the curves is not indicative of any physical behavior.

The results described above showed that run length increased if the motors were clustered rather than randomly spread over the surface of the cargo. We wondered if one cost of clustering might be to increase the time for the cargo to attach, compared to randomly placed motors. We performed simulations in which the cargo initially was resting on the microtubule and was subjected to thermal fluctuations that could cause it to diffuse away or to rotate randomly. In Figure 22 we compare the fraction of binding events (in which the motors attached to the microtubule) of cargos with motors randomly spread over the cargo surface to the case where the motors are clustered. One can see that clustering did not significantly affect the ability of the cargo to bind except for very large cargos at high viscosities (10 times that of water).

## Discussion

Our study of the effects of the cargo on transport has a number of ‘take-home’ messages. The first is that, at both the single-motor and multiple-motor levels, the presence of the cargo can significantly alter the effective ‘on’ rate/probability of successful binding of the motor(s) to the filament, because the center of mass of the cargo diffuses away from the microtubule relatively slowly, and while this is occurring, its rotational diffusion frequently brings the motor close enough to the microtubule to allow attachment. Thus, the cargo ‘helps’ the motor to attach, though the degree of assistance depends on cargo size and viscosity of the medium surrounding the cargo. Rapidly diffusing cargos might not linger long in the vicinity of a microtubule, but in a cell where there are multiple filaments available, these cargos could quickly find and bind to a filament.

Second, in order to for a motor to attach to the filament in a reasonable amount of time, the motor length needs to be longer or comparable to the radius of the cargo which may explain why motors are 60 to 110 nm in length.

Third, if motors are randomly arranged on the cargo's surface, the relationship between the number of motors present and the number of actually engaged motors depends strongly on the cargo size, so that different simple models of regulating cargo motion by recruiting motors to the cargo surface (either by a specified change in total number of motors, or by a specified change in local motor surface density) will have different effects on overall cargo motion as a function of cargo size. Thus, in order to have regulation affect a set of cargos equally, independent in variations in cargo size, it is best to have motors clustered in a small region on the cargo.

A further finding also supports the utility of motor clustering: for large cargos, if motors are randomly placed, achieving a reasonable number of engaged motors (n = 3–6) would require a large number of motors (50–100) to be present on the cargo, which appears inconsistent with biochemical characterizations of cargo-bound microtubule motors [4], though it is consistent with biochemical characterizations of cargo-bound myosin motors [18] which are likely randomly arranged on cargos [18], [19]. Overall, our findings suggest that, *in vivo*, microtubule motors are likely organized into clusters when present on large cargos, but that such clustering is unnecessary for small cargos.

In addition, a reasonable number of engaged motors would be required for long travel distances of several microns but not for short run lengths. Since microtubules can be tens of microns long compared to actin filaments which have a typical decay length of 1.6 microns [18], we expect long travel distances along microtubules but relatively short run lengths along actin filaments. Thus we predict the microtubule motors kinesin and dynein to be clustered on cargos while we expect the actin motor myosin V to bind randomly to cargos. There is clear experimental evidence for the random arrangement of myosin on cargos *in vivo*, and weak experimental evidence for the clustering of kinesin and cytoplasmic dynein [19].

For the purposes of this paper, we have assumed that the points where motors are attached to the cargos are fixed on the cargo's surface. This is true in some cases, e.g., when motors bind to dynactin which in turn binds to spectrin which is a filament that coats some vesicles [20], [21]. However, in other cases, the attachment points can diffuse through the fluid membrane of the vesicle and cluster at one location. An example of this is an experiment showing that motors dynamically accumulate at the tip of membrane tubes growing out of a vesicle as a consequence of the fluidity of the membrane [13], [22].

Clustering does not seem to affect the rate at which the first motor of a cargo attaches to a microtubule unless the cargo is large (greater than 200 nm) and the viscosity is high. Motor proteins are sufficiently long (greater than 50 nm) and rotational diffusion sufficiently rapid that the number of motors on a cargo does not significantly affect the rate at which the cargo binds to the microtubule.

## Supporting Information

Text S1 Details of Monte Carlo simulation of a cargo hauled by motor proteins along a microtubule.
(DOC)
