# Supplementary material for: How Molecular Motors Are Arranged on a Cargo Is Important for Vesicular Transport
Source: PLoS Comput Biol. 2011 May 5;7(5):e1002032. doi: 10.1371/journal.pcbi.1002032 (PMC3088656; doi:10.1371/journal.pcbi.1002032)
Supplement: Text S1 — Details of Monte Carlo simulation of a cargo hauled by motor proteins along a microtubule. (DOC) [file pcbi.1002032.s001.doc]

# Text S1: Details of Monte Carlo Simulation of a Cargo Hauled by Motor Proteins along a Microtubule

We outline the procedure for a three-dimensional Monte Carlo simulation of cargo travel toward the positive end of a cylindrical microtubule, as driven by multiple single-kinesin motors, which step along the microtubule by repeated attachment and detachment. The motor stepping action is the Monte Carlo simulation of a mechanoenzyme displacement that involves a steady-state Michaelis-Menten process of ATP binding to the motor head, unbinding from the motor head, and hydrolysis [1,2]. With regard to the cargo, the cargo is taken to be spherical, with radius R and density equal that of water near room temperature and atmospheric pressure. Cargo translational and rotational dynamics result from (i) forces and torques exerted by attached motors, (ii) random forces and torques exerted by the surrounding thermally fluctuating fluid medium, and (iii) an optionally applied external load acting on the center of mass of the cargo. The surrounding medium interacts with the cargo via Brownian motion in accordance with the forms of Stoke’s law applicable to both a translating and rotating sphere immersed in a fluid of dynamic viscosity η.

An overview of basic Monte Carlo simulation of cargo travel along a microtubule can be found in [2,3]. In what follows, we concentrate on describing those aspects of Monte Carlo simulation unique to three dimensions.

# Kinesin Simulation

Incremental displacement in the form of discrete steps d of a single-kinesin motor follows from repeated attachment to the microtubule and detachment from the microtubule of the kinesin motor head. This process involves a steady-state enzyme reaction that describes ATP binding to the motor head, unbinding from the motor head, and hydrolysis. The rate of this reaction, which we can denote as a velocity V when multiplied by d, is

| **Parameter** | **Value** |
| --- | --- |
| [ATP] | 2000 μM |
| d | 8 nm |
| k_on_ | 2 x 10^6^ M^-1^·sec^-1^ |
| k_0off_ | 55 sec^-1^ |
| k_cat_ | 105 sec^-1^ |
| k_attach_ | 5 sec^-1^ |
| k_back-detach_ | 2 sec^-1^ |
| d_1_ | 1.6 nm |
| δ_1_ | 1.3 nm |
| F_0_ | 8 pN |
| A | 107 |
| B | 0.029 μM |
| T | 300 K |
|  | |
| **Table S1.** Input parameters of kinesin simulation. | |

$\begin{matrix} V=\frac{V_{\max}[ATP]}{[ATP]+K_{M}}; & V_{\max}=k_{cat}d\left[1-\left(\frac{F}{F_{0}}\right)^{2}\right]; & K_{M}=\frac{k_{cat}+k_{off}}{k_{on}} \end{matrix}$. (1)

Here, [ATP] is the concentration of ATP; K_M_ is the Michaelis-Menten constant; k_on_, k_off_, and k_cat_ are the rates of binding, unbinding, and hydrolysis of ATP, respectively; F is the magnitude of a load on the motor directed toward the negative end of the microtubule; and F_0_ is the motor stall force. Additionally, the rate of unbinding k_off_ depends on the magnitude of the load; it is modeled as

, (2)

where k_0off_ is the chemical rate of unbinding (in the absence of load), d_1_ is the distance over which the work of the load is done toward unbinding the motor, T is the temperature, and k_B_ is the Boltzmann constant. Note that F implicitly includes the affect of thermally fluctuating cargo position and orientation due to the third law of motion.

A motor may exert a force and torque on the cargo, but only if its head is attached to the microtubule and its linkage is stretched. Attachment occurs at a rate defined by k_attach_. A motor head may in fact detach from a microtubule during the simulation with a probability that depends on the current state of the motor. Specifically, if the motor resides in the state that occurs just before ATP binding, then the probability per unit time of detachment from the microtubule is P_detach1_. If the motor resides in the state that occurs after ATP binding then the probability per unit time of detachment from the microtubule is P_detach2_. The two detachment probabilities per unit time may be expressed as

$\begin{matrix} P_{\det tach1}=\frac{BP_{step}}{[ATP]}; & P_{\det tach2}=\frac{P_{step}}{A}\exp\left(\frac{8}{F_{0}}\frac{F\delta_{l}}{k_{B}T}\right); & P_{step}=\frac{k_{cat}[ATP]}{[ATP]+K_{M}}\left[1-\left(\frac{F}{F_{0}}\right)^{2}\right] \end{matrix}$, (3)

where A is the maximum number of steps the motor head may take before detachment, B is a constant of proportionality, F_o_ is the stall force, and δ_l_ is the displacement associated with the work done by the load force. The probability per unit time of taking a step of length d is given by P_step_. The rate k_back-detach_ is the rate of detachment under a load equal to or higher than the stall force F_0_.

The single-kinesin process simulated in our Monte Carlo calculations is also described in detail in [2,3]. Table S1 summarizes the values of the input parameters that were used in our mechanoenzyme simulation. A detailed description of the Monte Carlo simulation algorithm follows later in this supplemental article.

# Cargo Simulation

The simulated motion of the cargo is governed by (i) the forces and torques exerted on it by kinesin motors that are attached to both its spherical surface and to the microtubule, (ii) the forces and torques exerted via random collisions of the cargo with thermally excited molecules of the surrounding fluid, and (iii) an external load that produces a force acting on the center of mass of the cargo, toward the negative end of the microtubule. On average, the net force and torque acting on the cargo are zero, i.e., on average the cargo is in mechanical equilibrium. However, at any given time step Δt of the Monte Carlo simulation, the cargo is subject to a stochastic net force and torque. Likewise, each motor that is attached to both a cargo and a microtubule experiences a stochastic load F, as introduced in (1), exerted by the cargo via tension on the stretched motor linkage.

## Cargo Translational Motion

At any time t, the forces acting on the spherical cargo of mass m and radius R, immersed in a fluid of dynamic viscosity η, satisfy the Langevin equation

. (4)

On the right side of (4) we see that the cargo is subject to a drag force proportional to its instantaneous velocity$\vec{v}\left(t\right)$, with constant of proportionality given by -α_T_, where α_T_ is the drag coefficient, i.e., Stokes’ law. Also appearing in (4) is , the sum of forces due to (i) an external load of magnitude F_L_ and (ii) the tension of N motors pulling on the cargo. This may be expressed as

, (5)

where $\hat{c}$ is a unit vector parallel to the microtubule, oriented toward its positive end, and k is a motor spring constant that we set to 0.32 pN/nm [2,4]. Also, is a vector whose tail resides at the point of attachment of the n-th motor to the sphere, with head positioned at the point of attachment of the n-th motor to the microtubule, where is the uncompressed length of the n-th motor. The sum of (5) is over the subset M of N that consists of motors whose spring forces are in an uncompressed state and attached to the microtubule. Figure S1 illustrates the position of a given motor on both the cargo and the microtubule.

Finally in (4), we also have the force$\vec{F}_{T}\left(t\right)$, which is the sum of all random thermally fluctuating forces acting on the cargo via molecules of the surrounding fluid. By construction , where the angle brackets refer to the averaging of these thermal fluctuations. With respect to this average the cargo is in mechanical equilibrium, which means that the average net force acting on the cargo is zero, such that the average velocity of the cargo is time independent. Thus, if we take the average of (4) and solve for the average velocity, we obtain the time-independent result. Hence, the average displacement of the cargo over a small time step Δt is given by

. (6)

With this in mind, we wish to integrate equation (4) in the presence of thermal fluctuations to obtain an expression for the displacement $\vec{x}\left(t\right)$ of the cargo over the time step Δt. In performing this integration we will assume the drag force to be slowly varying in comparison to the rapidly fluctuating thermal forces represented by$\vec{F}_{T}\left(t\right)$. In other words, the time scale associated with random collisions of fluid molecules with the cargo is smaller than any other time scale of the problem. This also implies that the time scale associated with the stepping action of the motors is greater than those of$\vec{F}_{T}\left(t\right)$. With these assumptions in place we may generalize (6), expressing the stochastic contribution in terms of a Cartesian vector $\vec{\varepsilon}=\left(\varepsilon_{x},\varepsilon_{y},\varepsilon_{z}\right)$, of the laboratory frame of reference, consisting of components that are random variates, viz.

. (7)

Here, $\sigma_{T}\vec{\varepsilon}$ is the random displacement that follows from the randomly fluctuating force$\vec{F}_{T}\left(t\right)$, where the standard deviation$\sigma_{T}$ is yet to be determined. If we choose the components of $\vec{\varepsilon}$ to be independent random variates drawn on a normal distribution, with $\left\langle\varepsilon_{i}\right\rangle=0$ and$\left\langle\varepsilon_{i}\varepsilon_{j}\right\rangle=\delta_{i,j}$, $i,j=x,y,z$, then from (7) we may write

. (8)

We now want to integrate (4) and reconcile it with (7) and (8). Consider (4) in Cartesian component form, multiply each equation by its component change in displacement $\Delta x_{i}\left(t\right)=x_{i}\left(t+\Delta t\right)-x_{i}\left(t\right)$, and take the average. The result is

. (9)

With , we can write (9) as

. (10)

Via the equipartition theorem we have. Also, we can assume if we take to be slowly varying such that and $\left\langle\Delta x_{i}\left(t\right)\right\rangle=0$. In this way, (10) becomes

, (11)

which has the solution

, (12)

where C is a constant of integration.

Now in (12), in the limit of the random walk, one has $\alpha_{T}t/m>>1$, which means we can neglect the term proportional to C. Since we then have

$\frac{d}{dt}\left\langle\Delta x_{i}\left(t\right)^{2}\right\rangle\cong\frac{2k_{B}T}{\alpha_{T}}$, (13)

which yields

$\left\langle\Delta x_{i}\left(t\right)^{2}\right\rangle\cong\frac{2k_{B}T}{\alpha_{T}}\Delta t$. (14)

The first term on the right of (8) is second order in Δt while the term on the right of (14) is first order in Δt. Thus, we can conclude that $\sigma_{T}^{2}=\left(2k_{B}T/\alpha_{T}\right)\Delta t$ is the more significant first-order term, which reconciles (14) with (7) and (8). Hence, we may write the stochastic process for translational motion of the cargo as

, (15)

where $\vec{x}(t)$ is the position of the center of mass of the cargo at time t, α_T_ is the coefficient of force associated with thermal fluctuations, σ_T_ is the standard deviation of a normal distribution, and $\vec{\varepsilon}=\left(\varepsilon_{x},\varepsilon_{y},\varepsilon_{z}\right)$ is a vector in Cartesian coordinates of the laboratory frame of reference that represents three independent random variates drawn on a normal distribution having zero mean and unit standard deviation.

In Monte Carlo simulation, to compute $\vec{x}(t+\Delta t)$ of (15), we must first obtain the ensemble of outcomes so that we may determine the mean force . Since it is easier to replace with when computing $\vec{x}(t+\Delta t)$ since, in this way, one does not have to save the history of outcomes at any given time t. This is what we do in practice.

## Cargo Rotational Motion

The stochastic process of rotational motion follows analogously to that of translational motion. The analog of (4) is the torque equation of the laboratory frame of reference given by

, (16)

where I = 2mR^2^/5 is the moment of inertia of a solid sphere and α_R_ is the drag coefficient of a drag torque proportional to angular velocity $\vec{\Omega}\left(t\right)$. The torque exerted on the cargo by attached motors, referenced from the center of mass of the cargo, can be expressed as

, (17)

where denotes the point of attachment of the n-th motor to the cargo, as illustrated in Fig. S1. Note that there is no torque due to the external load since it is applied to the center of mass of the cargo.

Analogous to the rapidly varying random force $\vec{F}_{T}\left(t\right)$ there is a rapidly varying random torque $\vec{N}_{R}\left(t\right)$ that enters (16). As in the case of $\vec{F}_{T}\left(t\right)$ we have by construction that . Again, with respect to this average the cargo is in mechanical equilibrium, which means that the average net torque acting on the cargo is zero, such that the average angular velocity of the cargo is time independent. Thus, if we take the average of (16) and solve for the average angular velocity we obtain the time-independent result .

Following the convention of Landau and Lifshitz [5], we introduce the Euler angles $\theta\left(t\right),\varphi\left(t\right),\psi\left(t\right)$ that describe the orientation of the spherical cargo at time t, as illustrated in Fig. S2. These angles can be used to define the angular displacement of the cargo, as well as a rotational transformation $R=R\left[\theta\left(t\right),\varphi\left(t\right),\psi\left(t\right)\right]$ between body coordinates 1,2,3 of the sphere and the Cartesian coordinates x,y,z of the laboratory frame of reference. The rotational transformation can be written as

. (18)

From a geometrical analysis, the angular velocity, expressed within the representation of body coordinates, is

. (19)

Applying the inverse of (18) to (19) gives the angular velocity in the representation of the laboratory frame, viz.

. (20)

Solving for in (20) we find

. (21)

Returning to our result , we assume the random variations of the Euler angles $\theta\left(t\right),\varphi\left(t\right),\psi\left(t\right)$ to be independent. Thus, for example, we have$\left\langle\Omega_{y}\left(t\right)\cos\varphi\left(t\right)\cot\theta\left(t\right)\right\rangle\cong\left\langle\Omega_{y}\left(t\right)\right\rangle\left\langle\cos\varphi\left(t\right)\right\rangle\left\langle\cot\theta\left(t\right)\right\rangle$, and analogous to the case of translational motion, we therefore introduce independent random variates $\varepsilon_{\theta},\varepsilon_{\varphi},\varepsilon_{\psi}$ to describe stochastic angular displacement. These stochastic terms have origins in the rapidly varying random torque $\vec{N}_{R}\left(t\right)$ of (16). In this way, analogous to (7), we integrate (21) over a small time step Δt as

, (22)

where $\varepsilon_{\theta},\varepsilon_{\varphi},\varepsilon_{\psi}$ are independent variates drawn on a normal distribution, with mean of zero and standard deviation of unity, as in the translational-motion case. To find σ_R_ we follow a procedure analogous to the one we outlined for the translational-motion case. Here, we simply quote the result, which is a standard deviation equal to

$\begin{matrix} \sigma_{R}=\sqrt{\frac{2k_{B}T}{\alpha_{R}}\Delta t}; & \alpha_{R}=8\pi\eta R^{3} \end{matrix}$. (23)

Equation (22) is our stochastic process of rotational motion.

In our Monte Carlo simulation of rotational motion, as in the case of translation motion, our practice is to replace with in (22), to facilitate ease of computation. We also replace the averages of trigonometric functions of the Euler angles with their instantaneous, stochastic values at time t. A description of the complete Monte Carlo simulation is given below.

## Initial Boundary Condition

We carried out Monte Carlo simulations over a set of scenarios using (15) and (22), with averages replaced by their instantaneous, stochastic counterparts, from which we calculated mean properties such as cargo run length and average velocity. Each scenario was subject to an initial boundary condition, which involved initially placing the cargo above the microtubule such that its closest point was z_0_ from the surface of the microtubule. A detailed description of the initial boundary condition follows, with Fig. S3 serving as illustration.

We defined a region of surface area of the spherical cargo on which we randomly attached motors. This area can be described by a cone with apex A at the center of the cargo and base projected toward the microtubule. A line AB extends from the apex through the center of the base to a point on and normal to the surface of the microtubule. This line, together with any side edge of the cone, is subtended by an angle ϕ, which we refer to as the cluster angle. The cluster angle ϕ defines, in 360-degree rotation about AB, the area of the cargo to which random attachment of motors can be performed. This region is boldly drawn in Fig. S3. The cluster angle can be varied between zero (attachment made only at the bottom point of the sphere) to a full 180 degrees (attachment made randomly to any point on the surface of the sphere).

Thus, for a given cluster angle, N motors may be randomly attached to the cargo of Fig. S3. The probability of attachment is uniform as a function of solid angle. Once all motors are attached, each motor linkage is placed in a relaxed, uncompressed state, directed radially outward from its point of attachment to the surface of the sphere. Each relaxed linkage, of length , is allowed to rotate along its point of attachment toward the line AB that is normal to the microtubule (and subtended by the cluster angle). If the linkage is capable of contacting the surface of the microtubule, of radius R_MT_, then the point of contact becomes the point of placement of the motor head, which typically involves compressing the linkage. Motors attached to the microtubule in this way do not exert force or torque on the cargo. In the event that no motors can attach to the microtubule, the cargo is rotated until the point of attachment of one motor to the cargo reaches the minimum distance to the microtubule, i.e., the point of attachment is directly over the microtubule. This ensures that a second attempt to attach motors to the cargo will result in at least one successful attachment to the microtubule.

A caveat of the attachment procedure is that a motor head must not be allowed to attach to the microtubule if attachment means that the motor linkage passes through the volume of the cargo. This constraint is often violated by motors with attachment to the portion of the cargo surface facing away from the microtubule, as in Fig. S4. This constraint is maintained throughout Monte Carlo simulation of cargo motion, not just during the motor attachment to the microtubule. In addition, the motor is not allowed to enter the microtubule.

Note that the procedure described above also is applied to motor attachment during cargo travel, not just during the establishment of the initial boundary condition. In this later case, as the cargo is traveling along the microtubule, a motor head can be attached or reattached by swinging the relaxed linkage toward the line AB, again starting with the motor head placed radially outward from its point of attachment to the cargo surface. The difference between attachment during travel and attachment as part of the initial boundary condition is that the line AB, while still normal to the microtubule surface, typically no longer bisects the cone of apex A. This is because the cargo, whilst traveling, has rotated way from the initial orientation depicted in Fig. S3. Thus, even when the cluster angle assumes the value ϕ = 0, the constraint of Fig. S4 may prevent motor reattachment in some situations.

When constructing the initial boundary condition, once all possible attachments to the microtubule have been made, the cargo is placed in mechanical equilibrium by adjusting the position of the cargo center of mass. This is typically necessary when an external load is to be applied. Since, generally speaking, motors may exert a net component of force on the cargo in the direction normal to the microtubule surface, the cargo is brought in contact with the microtubule surface to ensure these components are canceled by the normal force exerted by the microtubule on the cargo. If a non-zero external load is applied, then the cargo can be translated and rotated along the microtubule surface until mechanical equilibrium is satisfied. Keep in mind that motors only exert a force on the cargo if they are stretched; compressed motor linkages do not exert a force.

|  |  |
| --- | --- |
| **Parameter** | **Value** |
| η/η_water_ | 1 to 10 |
| z_0_ | 15 nm |
| R_MT_ | 12.5 nm |
| R | 50 nm to 500 nm |
|  | 110 nm |
| ϕ | 0^0^ to 180^0^ |
|  | |
| **Table S2.** Input parameters of cargo simulation. | |
|  | |

Once mechanical equilibrium is established, a set of Monte Carlo scenarios may be performed using this specific initial condition in order to simulate travel of the cargo down the microtubule. Since the initial boundary condition is stochastic, an ensemble of such boundary conditions must be obtained, representative of a specific cluster angle; for each member of the ensemble, a set of scenarios is simulated involving travel of the cargo down the microtubule. Table S2 summarizes input parameters used specifically for the cargo simulation. A description of the complete Monte Carlo simulation is given below.

## Monte Carlo Simulation Algorithm

The algorithm described here simulates the travel along a microtubule by a three-dimensional, spherical cargo immersed in a fluid, subject to Stokes’ law. The simulation is carried out by employing three distinct stochastic processes. These processes include (i) the process of kinesin motor stepping, ultimately defined by (1) via the underlying dynamics of motor-head attachment to the microtubule and detachment from the microtubule, accompanied by binding, unbinding, and hydrolysis of ATP; (ii) the translational Brownian motion of the cargo that follows from the process of (15); and the rotational Brownian motion of the cargo that follows from the process of (22). Each of these processes influences the propagation of the cargo toward the positive end of the microtubule.

Below we outline the steps of the algorithm:

*Simulation Start:*

1. For a given external load, N motors are randomly attached to the cargo within the region of the specified cluster angle.

   *New Initial Boundary Condition:*
2. With the cargo suspended above the microtubule at a distance z_0_, as in Fig. S3, an attempt is made to attach each of the N motors to the microtubule, in the manner described earlier. A motor cannot attach if its linkage passes through the volume of the cargo, as in Fig. S4. If no motors can be attached, then the cargo is rotated so that one of the attachment points of a motor to the cargo is directly under the microtubule. As long as the relaxed length of this motor’s linkage is at least z_0_, then at least one motor is guaranteed to attach to the microtubule.

   *New Cargo Travel Scenario:*
3. With the initial boundary condition established, such that a subset of N motors is now attached to the microtubule, a travel scenario is constructed by partitioning the passage of time into equal intervals Δt, starting from the initial time.

   *New Scenario Time Step:*
4. At a given interval of time, the following steps are taken:
   1. The instantaneous net force and torque acting on the cargo is computed.
   2. Each of the N motors is allowed to follow the process of stepping toward the positive end of the microtubule. The process followed by a motor may be described as follows:
      1. If the motor is detached from the microtubule, then a test is made to attach the motor, according to the probability of attaching $k_{attach}\Delta t$. The test is made by drawing a random variate from the uniform distribution, on the interval [0,1], and comparing it to the stated probability. If the test succeeds, which means the variate is less than the probability, then the motor is attached using the attachment algorithm described earlier, which completes the process.
      2. If the motor is attached then the following tests may occur:
         1. A test is made to see if the motor can detach from the microtubule according to either of the probabilities $P_{\det tach\text{ }1}\Delta t$, $P_{\det tach\text{ }2}\Delta t$, as computed via (3). If the test succeeds for either mode of detachment, then the motor is detached from the microtubule, which completes the process.
         2. If the test fails, then activating or deactivating the motor, i.e., ATP binding or unbinding, is implemented:
            1. If ATP is bound, then a test is made to determine if ATP is released using the probability $k_{off}\Delta t$, via (2). If the test succeeds, then ATP is released, which completes the process.
            2. If the head has no ATP, then a test is made to determine if ATP will bind using the probability $k_{on}\left[ATP\right]\Delta t$. If the test fails, such that there is still no ATP bound, then an additional test is performed to determine if the motor has stalled, i.e., whether the load F exceeds the stall force F_0_. If the load exceeds the stall force then a random variate drawn on the interval [0,1) is tested against the probability $k_{back-\det ach}\Delta t$ to simulate the possibility of the motor detaching from the microtubule under a stalling load.
         3. To reach this step, the ATP must be bound to the head, either because ATP attached before the process started or because it attached during the process of step 2, above. Since the motor is active, a test of ATP hydrolysis can be made using the probability of $k_{cat}\Delta t$. If this hydrolysis test succeeds then the motor is switched off. If the test fails then an additional test for detachment is made. The detachment test is the same as described in 2b, above. If this additional test succeeds then the motor is detached from the microtubule, which completes the process.
         4. If the process continues to this point then a test is made to see if the motor may take a step along the microtubule. The test can be made against the probability $\varepsilon$, where $\varepsilon=1-\left(F/F_{0}\right)^{2}$ is the efficiency function. If the test succeeds then the motor may make a step provided its linkage does not pass through the volume of the cargo. If the test fails then a detachment test like that of 2b is performed.
   3. The center of mass of the cargo is translated via (15) using the net force computed in step a. This involves drawing a normally distributed variate corresponding to each of the three Cartesian coordinates.
   4. The cargo is rotated via (22) using the net torque computed in step a and the instantaneous Euler angles. This also involves drawing a normally distributed variate corresponding to each of the three Euler angles.
5. Step 4 is repeated until all N motors are detached from the microtubule, at which point the travel scenario has ended. The final time step determines how far the cargo has traveled, i.e., the run length of the cargo for this scenario.
6. Step 3 is repeated for a specified number of travel scenarios, using the same initial condition.
7. Step 2 is repeated for a specified number of microtubule-attachment scenarios. Each invocation of step 2 produces a new initial boundary condition.

|  |
| --- |
|  |

1. Schnitzer MJ, Visscher K, Block SM (2000) Force production by single kinesin motors. Nat Cell Biol 2: 718-723.

2. Kunwar A, Vershinin M, Xu J, Gross SP (2008) Stepping, strain gating, and an unexpected force-velocity curve for multiple-motor-based transport. Curr Biol 18: 1173-1183.

3. Singh MP, Mallik R, Gross SP, Yu CC (2005) Monte Carlo modeling of single-molecule cytoplasmic dynein. Proc Natl Acad Sci U S A 102: 12059-12064.

4. Coppin CM, Pierce DW, Hsu L, Vale RD (1997) The load dependence of kinesin's mechanical cycle. Proc Natl Acad Sci U S A 94: 8539-8544.

5. Landau LD, Lifshitz EM (1976) Mechanics. Oxford: Pergamon Press, p. 110.
